# Supplementary material for: Proposal of MUAC as a fast tool to monitor pregnancy nutritional status: results from a cohort study in Brazil
Source: BMJ Open. 2021 May 24;11(5):e047463. doi: 10.1136/bmjopen-2020-047463 (PMC8149442; doi:10.1136/bmjopen-2020-047463)
Supplement: Author's manuscript [file bmjopen-2020-047463.draft_revisions.pdf]

BMJ Open is committed to open peer review. As part of this commitment we make the peer review history of every article we publish publicly available.

When an article is published we post the peer reviewers' comments and the authors' responses online. We also post the versions of the paper that were used during peer review. These are the versions that the peer review comments apply to.

The versions of the paper that follow are the versions that were submitted during the peer review process. They are not the versions of record or the final published versions. They should not be cited or distributed as the published version of this manuscript.

BMJ Open is an open access journal and the full, final, typeset and author-corrected version of record of the manuscript is available on our site with no access controls, subscription charges or pay-per-view fees (http://bmjopen.bmj.com).

If you have any questions on BMJ Open's open peer review process please email info.bmjopen@bmj.com

A proposal of a fast tool to monitor pregnancy nutritional status

Journal:	*BMJ Open*
Manuscript ID	bmjopen-2020-047463
Article Type:	Original research
Date Submitted by the Author:	03-Dec-2020

Complete List of Authors:	Miele, Maria; State University of Campinas Faculty of Medical Sciences, Obstetrics and Gynecology<br>Souza, Renato; Universidade Estadual de Campinas, Obstetrics and Gynecology<br>Calderon, IMP; UNESP Campus de Botucatu, Department of Gynecology and Obstetrics<br>Feitosa, Francisco; Federal University of Ceara, Obstetric Department of MEAC<br>Leite, Debora; Federal University of Pernambuco, Obstetrics and Gynecology<br>Rocha Filho, Edilberto; Federal University of Pernambuco, Obstetrics and Gynecology<br>Vettorazzi, Janete; Federal University of Rio Grande do Sul, Obstetrics and Gynecology<br>Mayrink, Jussara; Universidade Estadual de Campinas Faculdade de Ciencias Medicas, Obstetrics and Gynecology<br>Fernandes, Karayna; State University of Campinas Faculty of Medical Sciences, Obstetrics and Gynecology<br>Vieira, Matias; King's College London School of Life Course Sciences, Division of Women's Health<br>Pacagnella, Rodolfo; State University of Campinas, Obstetrics and Gynecology<br>Cecatti, Jose; State University of Campinas Faculty of Medical Sciences, Obstetrics and Gynecology

Keywords:	Maternal medicine < OBSTETRICS, NUTRITION & DIETETICS, Prenatal diagnosis < OBSTETRICS

I, the Submitting Author has the right to grant and does grant on behalf of all authors of the Work (as defined in the below author licence), an exclusive licence and/or a non-exclusive licence for contributions from authors who are: i) UK Crown employees; ii) where BMJ has agreed a CC-BY licence shall apply, and/or iii) in accordance with the terms applicable for US Federal Government officers or employees acting as part of their official duties; on a worldwide, perpetual, irrevocable, royalty-free basis to BMJ Publishing Group Ltd ("BMJ") its licensees and where the relevant Journal is co-owned by BMJ to the co-owners of the Journal, to publish the Work in this journal and any other BMJ products and to exploit all rights, as set out in our licence.

The Submitting Author accepts and understands that any supply made under these terms is made by BMJ to the Submitting Author unless you are acting as an employee on behalf of your employer or a postgraduate student of an affiliated institution which is paying any applicable article publishing charge ("APC") for Open Access articles. Where the Submitting Author wishes to make the Work available on an Open Access basis (and intends to pay the relevant APC), the terms of reuse of such Open Access shall be governed by a Creative Commons licence – details of these licences and which Creative Commons licence will apply to this Work are set out in our licence referred to above.

Other than as permitted in any relevant BMJ Author's Self Archiving Policies, I confirm this Work has not been accepted for publication elsewhere, is not being considered for publication elsewhere and does not duplicate material already published. I confirm all authors consent to publication of this Work and authorise the granting of this licence.

For peer review only

ORIGINAL RESEARCH - Miele.M.J.O et al

A proposal of a fast tool to monitor pregnancy nutritional status

Maria J O Miele[1]

Renato T Souza[1]

Iracema M P Calderon[2]

Francisco E Feitosa[3]

Débora F Leite[1,4]

Edilberto A Rocha Filho[4]

Janete Vettorazzi[5]

Jussara Mayrink[1]

Karayna G Fernandes[1]

Matias C Vieira[1,6]

Rodolfo C Pacagnella[1]

José G. Cecatti[1]

[1] Department of Obstetrics and Gynecology, University of Campinas (UNICAMP), School of Medicine, Campinas, SP, Brazil; [2] Department of Gynecology and Obstetrics, Botucatu Medical School, Sao Paulo State University (Unesp), Botucatu, SP, Brazil;[3] Obstetric Department of MEAC - Maternity School of the Federal University of Ceará, Fortaleza, CE, Brazil; [4] Department of Maternal and Child Health, Federal University of Pernambuco, Recife, PE, Brazil; [5] Department of Obstetrics and Gynecology, Maternity Hospital, Federal University of RS, Porto Alegre, RS, Brazil; [6] Division of Women and Children's Health, School of Life Course Sciences, Faculty of Life Sciences and Medicine, London, United Kingdom.

Correspondence: José Guilherme Cecatti

Department of Obstetrics and Gynecology, State University of Campinas

R. Alexander Fleming, 101 - Cidade Universitária, Zip Code 13081-970 - Campinas, SP – Brazil

Tel 55.19.35219482

Fax 55.19.35219304

For peer review only

Email : cecatti@unicamp.br

Abstract:

Assessment of maternal nutritional status and early identification of risk factors can provide an opportunity to avoid health problems for women and their offspring. In Brazil, the monitoring is by calculating Body Mass Index (BMI) using curves categorized with nutritional status. However, BMI curves depend on followed measures in settings adequately equipped and women wearing fewer clothes for best accuracy. Mid-Upper Arm Circumference (MUAC) is another biological variable identified as a tool for rapid assessment of nutritional status that is correlated with BMI.

Purpose: We aim to surrogate BMI by MUAC cut-offs for rapid screening of maternal nutritional status starting at mid-pregnancy.

Patients and methods: The multicenter cohort study entitled "Preterm SAMBA" enrolled 1,165 pregnant women from 2015 to 2018 with diverse ethnic characteristics from different Brazilian regions at 3 prenatal care visits.

Results: A correlation between MUAC and BMI was performed, with significant positive associations in three set points analyzed (rho: 0.872, 0.870, and 0.831, respectively). Based on BMI categories of nutritional status, we estimated the best MUAC cut-off points, finding measures according to each category: underweight <25.75cm for three gestational weeks analyzed; overweight 28.11 - 30.15cm (19-21w), 28.71 - 30.60cm (27-29w), and 29.46 - 30.25cm (37-39 w); and obese >30.15cm (19-21w), >30.60cm (27-29w) and >30.25cm (37-39w) per gestational week. Therefore, we defined as adequate between 25.75 - 28.10cm (19-21w), 25.75 - 28.70cm (27-29w), and 25.75 - 29.45cm (37-39w) of MUAC.

Conclusion: We conclude that MUAC can be useful as a surrogate for BMI as a faster screening of nutritional status in pregnant women

Keywords: Mid-upper arm circumference; pregnancy; Body Mass Index; nutritional status; malnutrition; Risk Factors; Pregnancy Outcome.

For peer review only - http://bmjopen.bmj.com/site/about/guidelines.xhtml

Strengths and limitations of this study

- This study was a multicenter cohort performed in different regions of the country and exploring changes in maternal body composition.
- The assessment was standardized carried out by maternity health teams and included in the system in real time.
- Although the sample size is robust, the original study was not specifically designed for this assessment, being an analysis of secondary objectives

For peer review only

Introduction

Antenatal care represents a window of opportunity to promote healthy life habits, provide individualized care and prevent adverse health outcomes [1]. Worldwide, the concern about obesity can be extended to maternal underweight and malnutrition, with short and long-term consequences [2]. The Institute of Medicine (IOM) developed a guideline for American pregnant women to monitor maternal weight gain based on the World Health Organization (WHO) body mass index classification (BMI)[3]. In Brazil, the health system monitors the risk for malnutrition by BMI Atalah curves according to gestational week [4]. However, it is necessary to measure maternal weight and height early in pregnancy. In the absence of this information, self-reported weight should be used [5]. In low or middle-income countries, it is common for women to seek antenatal care very late. It is also not uncommon for women to skip antenatal care altogether, resulting in missing information on weight before or during pregnancy. As a result, monitoring of nutritional status by BMI becomes less effective [6–8]. Moreover, there is a tendency to underestimate weight and overestimate height when measures are dependent on maternal recall. Anthropometric measurements are frequently not recorded in the prenatal booklet, compromising the accuracy of nutritional status assessment [9–11].

Mid-Upper Arm Circumference (MUAC) has been recognized as a fast tool adopted to monitor nutritional status, and it is strongly correlated with BMI [12]. It has already been implemented for adult care in under-resourced settings, and for monitoring maternal

For peer review only - http://bmjopen.bmj.com/site/about/guidelines.xhtml

undernourishment and fetal growth [13–15].Thus, MUAC allows the assessment of protein intake and storage, related to severe undernutrition [16]. Despite the global obesity pandemic coexisting with undernutrition, there is a maternal and fetal health risk of developing non-communicable diseases such as hypertension, diabetes, and delivery of preterm small-for-gestational age (SGA) neonates, compromising the health of the future offspring [17,18]. Poverty and malnutrition are strongly associated with low birthweight and stunted growth in the first 1000 days of life [18–21]. Brazil has a different demographic density, with higher densities in the South/Southeast regions and Northeast coastal compared to the Northern and Central-West regions with larges empty spaces occupied by the Amazon rainforest and turned to agriculture and livestock[22,23]. In this country with socioeconomic disparities, we have an opportunity to investigate in a representative sample an option to faster screening risk of maternal nutritional status from mid-pregnancy onwards, allowing risk assessment related to abnormal nutrition, which may affects pregnancy outcomes. Implementing an easy and reproducible tool to monitor nutritional status is a great opportunity for scaling-up risk assessment related to abnormal and suboptimal nutritional conditions, monitor pregnancy nutritional interventions, and facilitate the provision of a more equitable antenatal care.

Material and methods

Study design

This study corresponds to a secondary objective of the multicenter cohort study entitled "Preterm SAMBA - Preterm Screening and Metabolomics in Brazil and Auckland," including 1,165 Brazilian nulliparous women with singletons, and no history of previous severe clinical condition. Detailed information on research methods and procedures of this study has already been fully explored and published elsewhere [24,25]. Data were collected between 2015 and 2018 at five Brazilian hospitals located in three different geographical regions that have distinctive physical and ethnic characteristics (CAISM at the University of Campinas and Maternity Hospital from Botucatu Medical School in the Southeast; Maternity of the Hospital of Clinics, Federal University of Rio Grande do Sul in the South; and Clinics Hospital, Federal University of Pernambuco and Maternity School Assis Chateaubriand of the Federal University of Ceará in the Northeast). Pregnant

For peer review only - http://bmjopen.bmj.com/site/about/guidelines.xhtml

women receiving routine prenatal care who met the inclusion criteria were included in the study between 19 and 21 weeks of gestation for the first study interview, in which data collection and anthropometric measurements were recorded (Figure 1).

Study procedures

The coordinating center for this study was the University of Campinas (CAISM) where the first research meeting took place with health professionals from each center. These professionals were trained by a nutritionist to take anthropometric measurements in pregnant women. Anthropometric measurements were obtained at three set points: 19-21, 27-29 and 37-39 weeks. We excluded from the analysis data from women with stillbirth, preterm birth, and from those considered as loss of follow up. All centers inserted maternal anthropometry data into the database of the study (MedSciNet® AB, Sweden). Height (cm) and weight (kg) were measured by a stadiometer and weighing scale, with the feet close to the heels, buttocks, and shoulder blades aligned and the head positioned in the horizontal plane of Frankfurt. From the results of height and weight, an automatic estimate of Body Mass Index (BMI) was generated by software using the formula: weight/height^2. The Mid-Upper Arm Circumference (MUAC) was measured with a flexible non-extensible tape. The left arm circumference was measured in centimeters at the mid-point between the acromion and shoulder by trained health professionals using a standard scale. All MUAC anthropometric measurements were performed three times and the mean was recorded. Two researchers checked all MUAC results. When the average variation was considered a deviation from the mean of the remaining values from the same women, the mean of the two closest recorded measures was used. BMI values were categorized by the Atalah curve[4], based on the Brazilian Ministry of Health´s reference to monitor pregnancy[26], identify adult maternal nutritional status and develop MUAC cut-off points. According to this criterion, the adequacy of Atalah's classification determined that there was no risk for malnutrition and not the adequacy of other classifications. This instrument was used to identify nutritional status in pregnant women according to BMI and develop MUAC cut-off points.

For peer review only - http://bmjopen.bmj.com/site/about/guidelines.xhtml

Statistical analysis

To compare BMI, MUAC, and maternal weight measurements and investigate whether differences in values exist for the measures, a repeated measures one-way ANOVA test was used. Statistical significance was determined using the Bonferroni's multiple comparisons test, with alpha = 0.05. Comparisons were made between three set points (at different gestational ages). For categorical variables, chi-square tests were performed to analyze differences between BMI classification at each set point. Each row was analyzed individually, without assuming a consistent SD. Then, a post hoc test was run to assess the differences between all values and find the result of each comparison, using Dunn's multiple comparisons test. Histograms of maternal MUAC and BMI were constructed to determine the distribution of each measurement for each set point separately and for all datasets combined. The Normal QQ plot Normality and Log normality tests were applied using the Shapiro-Wilk test and the Kolmogorov-Smirnov test to compare normal and lognormal distributions. Spearman's correlation coefficient was applied to estimate the linear relationship between MUAC and BMI. Afterwards, the Receiver Operating Characteristic (ROC) curve was plotted to obtain the performance of MUAC measures to predict maternal BMI for all possible cut-off points. The area under the curve (AUC) was calculated along with its 95% confidence interval (CI). Based on coordinates of the curve, the most accurate MUAC cut-off point was selected for the MUAC variable. For each BMI cutoff, we calculated a 2x2 table showing the cross-tabulation of MUAC measurement and calculated accuracy using McNemar's test of concordance (continuity corrected) [27]. To estimate sensitivity, specificity, Negative Predictive Value (NPV), and Positive Predictive Value (PPV), BMI was calculated for each gestational week considering normal the results from BMI adequacy classification. From this categorization, MUAC values were selected according to BMI references. The Youden method was calculated (highest sensitivity + specificity -1) to identify the best cut-off measures based on the largest vertical distance between the ROC curve and the diagonal curve, for each visit and separated for classification. The positive and negative Likelihood Ratio values was calculated according to Fletcher [28]. For the agreement between BMI and MUAC measurements, weighted Cohen's Kappa coefficient was used. Ordinal responses with disagreements were calculated according to their squared distance from the perfect agreement (squared weights)[29].

For peer review only - http://bmjopen.bmj.com/site/about/guidelines.xhtml

Ethics approval

The current study was approved by each local Institutional Review Board (IRB) and amended by the Brazilian National Committee for Ethics in Research (CONEP) - Letter of approval 1.048.565 issued on 28th April 2015. The study complies with national and international regulations for human being experiments, including resolution CNS 466/12 of the Brazilian National Heath Council and the 1989 Declaration of Helsinki. All women signed an informed consent form before enrolment. This study follows the Declaration of Strengthening Report on Observational Studies in Epidemiology[30].

Results

Out of a total of 1,165 nulliparous women (Figure 1), 461 (39.6%) were from the Southeast, 139 (11.9%) from the South and 565 (48.5%) from the Northeast of Brazil. Maternal age at the first visit corresponded to 291 (25.0%) women younger than 20 years (97 under age 17 years), 796 (68.3%) aged 20 to 34 years, and 78 (6.7%) older than 34 years. From the total sample, 578 (49.6%) women self-declared as having brown skin, 117 (10.0%) as having black skin, 462 (39.7%) as white skin and 7 (0.6%) as yellow, and 1 (0.1%) as having another ethnicity. Of this sample, 52 (4.5%) women lived with less than U$ 250 per month, 253 (21.7%) received from U$ 251 to 500; 380 (32.6%) women had a monthly income ranging from U$ 501 to 1,000 and 480 had an income over U$: 1,000 (41.2%).

Table 1 shows the distribution of anthropometric measurements, including the BMI of all participating women according to each set point. The mean variation in BMI measurements throughout pregnancy was greater than MUAC, and the opposite was observed in extreme measures (minimum and maximum), with greater variations in MUAC and lower BMI.

Figure 2 shows the correlation between BMI and MUAC according to different pregnancy set points. The Shapiro-Wilk normality was used to test whether data were different from normal distribution ($p<0.05$). Spearman's correlation coefficient was applied. BMI categories were represented by four different colors and dot size differentiated maternal weight (Figure 2).

To determine the most accurate MUAC value, different categories of nutritional status were selected by BMI classification obtained at three standard prenatal visits scheduled.

For peer review only - http://bmjopen.bmj.com/site/about/guidelines.xhtml

Differences were significant ($p<0.05$) for all categories and for three set points analyzed ($p<0.0001$). The Area Under the Curve (AUC) was statistically positive at three set points, and robustly significant for screening extreme nutritional status, notably for obese women (Figure 3).

The AUC showed that MUAC had a high discrimination capacity for nutritional status obtained by the actual gold standard method (BMI). Table 2 shows MUAC cutoff measures analyzed for use as a screening method for maternal nutritional status. At the first prenatal visit, accuracy was lower than at other gestational ages assessed. It was noteworthy, however, that a substantial difference existed between the predictive values, although the calculus of Likelihood (positive and negative) confirmed a slight probability of screening women at risk for malnutrition (obese, underweight, or overweight). There was no probability of screening for the lack of risk. The diagnostic test indicated that this measure was a good alternative to the screening test, especially for obese and underweight women. Measures between the cutoff for underweight and overweight were identified as within normal, or desirable. Afterwards, an interaction charter between MUAC and BMI classifications was constructed, using selected MUAC measures as a model for gestational age. It is possible to observe differences among the curves, with a better agreement for extremes of classification (obese and underweight). Figure 4 shows changes after the second set point or from the 27th week of gestation onwards, with a better relationship between categories. To compare the concordance between categories of two different methods of assessment, Cohen's Kappa statistic was applied, showing substantial agreement and reporting an excellent agreement beyond chance (Figure 4). Box 1 presents as a result of our proposal to monitor the maternal nutritional status by cut-offs for the MUAC tape with colors according to the classifications and degrees of risk

Discussion

This study investigated the hypothesis that the MUAC cutoff, at different stages of pregnancy, should be a feasible alternative to BMI and is a quicker tool for screening maternal nutritional status in Brazilian pregnant women. We confirmed a strong correlation between BMI and MUAC of Brazilian pregnant women at three set points covering distinct gestational weeks: mid pregnancy (19-21w), early third trimester (27-29w) and late pregnancy (37-39w). We achieved our goals of identifying MUAC cut-off values for different gestational weeks and malnutrition risk

For peer review only - http://bmjopen.bmj.com/site/about/guidelines.xhtml

levels and present a simple tool as an option to faster screening maternal nutritional status from mid-pregnancy to 39 weeks.

Our results are similar to findings obtained by studies that have tested this correlation at early and late gestational age, with diverse sociodemographic characteristics [6,31,32]. Most studies have associated MUAC with maternal malnutrition linked to underweight, obesity, and low birthweight (LBW). In addition, some authors have assessed MUAC in non-pregnant women and correlated those cutoffs with adult BMI in their own population [8,33–35].

Malnutrition during pregnancy increases the risk for low birthweight, preterm birth, and small or large for gestational age, among others. Therefore, MUAC has been used as an anthropometric measurement in routine antenatal care to detect malnutrition focused on low maternal weight in middle and low-income countries [36]. We identified that a single MUAC measure of (<25.75cm) is a predictor of underweight and poor nutritional risk. However, a literature review addressing which anthropometric values indicative of acute malnutrition were associated with adverse birth outcomes, proposed a cutoff point of 23cm to identify undernourished women. This value was considered to be correlated with lower pre-pregnancy BMI measures (<18.5kg/m^2) and the occurrence of adverse pregnancy outcomes. Our thresholds are in accordance with the Brazilian recommendation for BMI at mid-pregnancy. The reference value of the Health System for Brazilian pregnant women at 6 weeks of gestation is a BMI <19.9kg/m^2, close to the IOM recommendations of a BMI of <19.8 kg/m^2 [3,37].

Although MUAC is considered a stable measure, with less variation in comparison to BMI [17], it was clearly observed between both curves by the trace throughout pregnancy. There was a smooth MUAC curve contrasting to the increasing sharp trace of the BMI curve, with a significant agreement among assessments. Our results show not only changes in measures associated with poor nutritional status, but also a mild change in other classifications. A prior study with women in Argentina tested MUAC cut-off points using the curves generated in their second trimester of gestation to detect LBW. The authors indicated a slight difference in MUAC cut-offs for undernutrition status according to gestational week, as well as a MUAC of 24.5cm for pregnant women ≤16 weeks, <25.5cm at 28 weeks and <26.5cm at 36 weeks [6]. In India, MUAC was assessed in the first trimester to detect LBW, with an accuracy of 58.7, as well as positive and negative predictive values of 38.8% and 76.8%, respectively. The authors defined <22.5cm as

For peer review only - http://bmjopen.bmj.com/site/about/guidelines.xhtml

the best MUAC cutoff that alerts to an underweight measure and is a predictor of LBW in the first trimester [38]. High AUC values, in most results, show a good relationship between BMI and MUAC. The results showed a high degree of accuracy of MUAC cutoffs satisfactorily beating the current standard of classification for nutritional status (Table 2). In terms of correlations between values of MUAC, those results are similar to ours, showing a better capacity to find more women who are not at risk, but not missing any women at risk for malnutrition. Nevertheless, in the absence of a 'gold standard' for nutritional risk screening, an African study using a combination of validity tools to diagnose malnutrition found a significant association between MUAC, BMI and the Malnutrition Universal Screening Tool (MUST), searching for poor nutritional status in the adult population (men and women). However, BMI and MUST missed undernutrition in 38% and 43% patients, respectively, whereas MUAC was tracked in 100%. In South Africa, a MUAC value <23cm was adopted for severely underweight (BMI < 16 kg/m^2). A value of < 24 cm (BMI < 18.5 kg/m^2) was used to screen for malnutrition or a population at risk for malnutrition [39].

In our study, a MUAC value of 25.75 was used to identify low maternal weight (19 - 21 weeks), different from the value proposed in the literature, referring to early gestation or a different population. Although there was a better agreement among values between <22 and <23cm to identify LBW, a MUAC of <23cm is more highly recommended for the African and Asian contexts, noting that these values were not linked to gestational age [36]. The Khadivzadeh study with healthy women of reproductive age, indicated that a value <24cm was the best MUAC measure to detect underweight Iranian women [33]. A study in Spain with 1,373 male and female subjects of different ages indicated that a MUAC ≤22.5cm correlates more closely with BMI <18kg/m^2 [34].

The Guidelines for Maternity Care in South Africa monitor maternal nutritional status in antenatal screening using MUAC measures of <23cm, categorized as underweight. For the group of underweight women, this requires a search for infection and neoplasia. Fetal growth assessment and nutritional support are necessary [40]. A study on the effect of obesity, diet and preeclampsia conducted in Ethiopia adopt MUAC classification values <23cm for underweight, and between 23 and <25cm for adequate. Those authors observed that women with a MUAC <25cm had healthier habits and ate more vegetables and fruits. Along with the group of MUAC 21-23cm, those women have a three-fold lower chance of developing preeclampsia. Women with MUAC between 23–24.99cm were considered to have optimal status and <23cm was categorized

For peer review only - http://bmjopen.bmj.com/site/about/guidelines.xhtml

as underweight [17]. Similarly, our first cut off measure that is likely to determine diseases linked to overweight/obesity such as preeclampsia, starts at a MUAC of 28.11cm. It is important to highlight the difficulty in screening for disorders in pregnant women in the first trimester of pregnancy in some countries. The rate of the first antenatal care visit in the first trimester of pregnancy was estimated to be 58·6% worldwide, whereas it was 48·1% in developing countries and 24·0% in low-income countries[7].

For the conditions associated with excessive weight (overweight and obesity), South African Guides to maternal care classify a MUAC ≥30cm as obese. This measurement raises concern for hypertension, preeclampsia and gestational diabetes, guiding actions to prevent fetal macrosomia and complications during labor and delivery [40]. In Ethiopia, a MUAC ≥25cm is categorized as overweight and obese, where authors highlight that MUAC measurements between 28 and 39cm are more capable of predicting complications such as preeclampsia [17]. These parameters originated from a previous study in Zimbabwe with a black African population. Those authors found that women with a MUAC between 28 and 29 cm had a fourfold higher risk than those who had a MUAC between 21 and 23 cm [41].

From a sample of 2000 women, ranging from 15-45 years of age in the Islamic Republic of Iran with a BMI >29kg/m^2, it was determined that a MUAC measure >30.5cm can estimate BMI, detect nutritional disorders, and is useful to search for obesity [33]. Previously, a meta-analysis using data from individual participants in seven countries explored the potential to find a significant MUAC cutoff point to identify malnutrition in pregnant women. Mean MUAC varied widely between countries evaluated, as well as according to the weeks in which measurements were taken. In early pregnancy, measurements were between 21.8 and 23.0 cm. In the second and third trimester of pregnancy, averages were 25.2 and 26.5 cm. In the postpartum period, the average ranged from 27.1 to 28.9 cm. The authors concluded that MUAC cut-off values can vary widely internationally and were dependent on the population and time of assessment [12].

There seems to be a wide difference in MUAC according to the population, age of pregnant women, and gestational week for the underweight category (21 to 24cm), and particularly in the search for the overweight/obesity category (29.0cm to >33cm). We presumed that nutritional status was adequate at measurements between >24cm and <29cm. MUAC cut-off values found in the literature were commonly comparable to pre-pregnant BMI, with an

For peer review only - http://bmjopen.bmj.com/site/about/guidelines.xhtml

extremely small difference between those values and our results at mid-pregnancy. The consequences of extreme anthropometric measurements are linked to adverse outcomes for the mother and child. The socioeconomic, educational and ethnic diversity of the Brazilian population is tremendous, and these characteristics can influence maternal body composition [42].

MUAC must be specific to different populations. The originality of the study was to investigate a cohort of over a thousand pregnant women from different regions of Brazil. In the Brazilian pregnant population, there are no studies showing that MUAC is effective at screening risks for extreme cut-offs measures (low weight and overweight/obesity).

We assessed a tool capable of alerting to the risks of inadequate nutrition and their probable consequences linked by the levels of malnutrition, especially needed in settings were anthropometric measures are limited or missing. Once any measure indicating inadequacy is tracked, the need for investigation and eventual support of the diet and nutritional counseling, in addition to monitoring blood pressure and blood glucose, is highlighted.

A possible limitation is that this analysis was conducted as a secondary objective of the original study that had no specific sample size calculation. Therefore, the number of women included could be insufficient for all the associations assessed. Strengths include prior training of health professionals, real-time data inclusion, use of additional statistical procedures to explore results and mainly, a MUAC cutoff point defined for a Brazilian population of pregnant women (at three regions of the country, with different regional characteristics).

In conclusion, MUAC has the advantages over BMI due to simplicity of application. It does not require the calculation of other measures and is independent of pre-pregnancy weight recall. In a multiethnic population, our MUAC cut-off measures could be used as a faster screening tool of nutritional status in pregnant women. It may track risk and provide timely intervention to avoid adverse pregnancy outcomes. Still, more research will be welcome in order to validate these cutoffs for pregnancy outcomes.

Conclusion

In conclusion, MUAC has the advantages over BMI due to simplicity of application. It does not require the calculation of other measures and is independent of pre-pregnancy weight recall. In a multiethnic population, our MUAC cut-off measures could be used as a faster screening tool

For peer review only - http://bmjopen.bmj.com/site/about/guidelines.xhtml

of nutritional status in pregnant women. It may track risk and provide timely intervention to avoid adverse pregnancy outcomes. Still, more research will be welcome in order to validate these cutoffs for pregnancy outcomes.

Acknowledgments

The Preterm SAMBA study group also included: Maria L Costa, Mary A. Parpinelli, Rafael B Galvão, José Paulo Guida, Danielly S Santana, Bianca F. Cassettari, Daisy de Lucena, Denise F. Cordeiro, Elias F Melo Junior, Danilo Anacleto, Lucia Pfitscher, Luiza Brust.

This study was granted jointly by the Brazilian National Research Council (CNPq) (Award 401636/2013-5) and the Bill and Melinda Gates Foundation (grant OPP1107597). The funders played no role whatsoever in study design, writing of the manuscript or in the decision to submit the manuscript for publication.

Patient and public involvement

Patients or the public were not involved in the design, or conduct, or reporting, or dissemination plans of our research

Disclosure

The author reports no conflicts of interest in this work.

Data availability

Data are available upon reasonable request

Funding

This study was selected for sponsoring from the research call “Grand Challenges Brazil: Reducing the burden of preterm birth” number 05/2013 jointly issued by the Brazilian

For peer review only - http://bmjopen.bmj.com/site/about/guidelines.xhtml

358 National Research Council (CNPq) (Award 401636/2013-5) and the Bill and Melinda Gates Foundation (grant OPP1107597). The funders played no role whatsoever in the study design, writing of the manuscript nor in the decision to submit the manuscript for publication.

Authors contributions

MJM, RTS, JM, RCP, MCV and JGC designed the study; MJM, RTS, JM, IMC, FEF, DFL, EAR, KGF and JV conducted data collection; MJM, JGC and MCV conducted data analysis. All authors had access and participated in the interpretation of results. MJM wrote the first draft of the manuscript, reviewed initially by JGC and then by all authors who read, revised and approved the final version submitted for publication

References

1. World Health Organization. *WHO Recommendations on Antenatal Care for a Positive Pregnancy Experience*. Geneva, Switzerland; 2016.
2. Zanardo V, Mazza A, Parotto M, Scambia G, Straface G. Gestational weight gain and fetal growth in underweight women. *Ital J Pediatr*. 2016;42(1). doi:10.1186/s13052-016-0284-1
3. Rasmussen KM, Yaktine AL, Institute of Medicine (US), National Research Council (US), Committee to Reexamine IOM. *Weight Gain During Pregnancy: Reexamining the Guidelines*. Washington (DC); 2009.
4. Atalah E, Castillo C, Castro R, Aldea A. Proposal of a new standard for the nutritional assessment of pregnant women. *Rev Med Chil*. 1997;125:1429-1436.
5. BRASIL. Ministério da Saúde. Manual Orientador para Aquisição de Equipamentos Antropométricos. 2011:1-18.
6. López LB, Calvo EB, Poy MS, del Valle Balmaceda Y, Cámera K. Changes in skinfolds and mid-upper arm circumference during pregnancy in Argentine women. *Matern Child*

For peer review only - http://bmjopen.bmj.com/site/about/guidelines.xhtml

Nutr. 2011;7(3):253-262. doi:10.1111/j.1740-8709.2009.00237.x

7. Moller AB, Petzold M, Chou D, Say L. Early antenatal care visit: a systematic analysis of regional and global levels and trends of coverage from 1990 to 2013. *Lancet Glob Heal*. 2017;5(10):e977-e983. doi:10.1016/S2214-109X(17)30325-X

8. Das A, Saimala G, Reddy N, et al. Mid-upper arm circumference as a substitute of the body mass index for assessment of nutritional status among adult and adolescent females: learning from an impoverished Indian state. *Public Health*. 2020;179:68-75. doi:10.1016/j.puhe.2019.09.010

9. Mello LEA de M. Organização Geral do Sistema Nervoso. In: *Fisiologia Básica*. 2nd ed. Rio de Janeiro: Guanabara Koogan; 2017:131-166.

10. Morais SS, Ide M, Morgan AM, Surita FG. A novel body mass index reference range - an observational study. *Clin Sci*. 2017;11(9):698-707. doi:10.6061/clinics/2017(11)09

11. Natamba BK, Sanchez SE, Gelaye B, Williams MA. Concordance between self-reported pre-pregnancy body mass index (BMI) and BMI measured at the first prenatal study contact. *BMC Pregnancy Childbirth*. 2016;16(1):1-8. doi:10.1186/s12884-016-0983-z

12. Tang AM, Chung M, Dong K, et al. *Determining a Global Mid-Upper Arm Circumference Cutoff to Assess Malnutrition in Pregnant Women*. Washington, DC; 2016.

13. Petraro P, Madzorera I, Duggan CP, et al. Mid-arm muscle area and anthropometry predict low birth weight and poor pregnancy outcomes in Tanzanian women with HIV. *BMC Pregnancy Childbirth*. 2018;18(1). doi:10.1186/s12884-018-2136-z

14. Papathakis PC, Singh LN, Manary MJ. How maternal malnutrition affects linear growth and development in the offspring. *Mol Cell Endocrinol*. 2016;435:40-47. doi:10.1016/j.mce.2016.01.024

15. Kumar P, Sareen N, Agrawal S, Kathuria N, Yadav S, Sethi V. Screening maternal acute malnutrition using adult mid-upper arm circumference in resource-poor settings. *Indian J Community Med*. 2018;43(2):132-134. doi:10.4103/ijcm.IJCM_248_17

16. Sultana T, Karim MN, Ahmed T, Hossain MI. Assessment of Under Nutrition of Bangladeshi Adults Using Anthropometry: Can Body Mass Index Be Replaced by Mid-Upper-Arm-Circumference? *PLoS One*. 2015;10(4):e0121456. doi:10.1371/journal.pone.0121456

For peer review only - http://bmjopen.bmj.com/site/about/guidelines.xhtml

17. Endeshaw M, Abebe F, Worku S, Menber L, Assress M, Assefa M. Obesity in young age is a risk factor for preeclampsia : a facility based case-control study, northwest Ethiopia. *BMC Pregnancy Childbirth*. 2016;16(237). doi:10.1186/s12884-016-1029-2

18. Black RE, Victora CG, Walker SP, et al. Maternal and child undernutrition and overweight in low-income and middle-income countries. *Lancet*. 2013;382(9890):427-451. doi:10.1016/S0140-6736(13)60937-X

19. Maria A, Menezes B, Oliveira PD, et al. Associations between growth from birth to 18 years, intelligence, and schooling in a Brazilian cohort. *Am J Clin Nutr*. 2020;nqaa047. doi:10.1093/ajcn/nqaa047

20. Park S, Sappenfield WM, Bish C, Salihu H, Goodman D, Bensyl DM. Assessment of the Institute of Medicine recommendations for weight gain during pregnancy: Florida, 2004-2007. *Matern Child Heal J*. 2011;15(3):289-301. doi:10.1007/s10995-010-0596-5

21. Leary S, Fall C, Osmond C, et al. Geographical variation in relationships between parental body size and offspring phenotype at birth. *Acta Obstet Gynecol Scand*. 2006;85(9):1066-1079. doi:10.1080/00016340600697306

22. Instituto Brasileiro de Geografia e Estatistica- IBGE. Mapa de Densidade Demográfica de 2010. Densidade Demografica. https://censo2010.ibge.gov.br/noticias-censo.html?view=noticia&id=1&idnoticia=2501&busca=1&t=ibge-lanca-mapa-densidade-demografica-2010. Published 2010. Accessed September 1, 2020.

23. Instituto Brasileiro de Geografia e Estatistica- IBGE. Mapa densidade demográfica. Instituto Brasileiro de Geografia - IBGE. https://brasilemsintese.ibge.gov.br/territorio/densidade-demografica.html. Published 2010. Accessed September 1, 2020.

24. Cecatti JG, Souza RT, Sulek K, et al. Use of metabolomics for the identification and validation of clinical biomarkers for preterm birth: Preterm SAMBA. *BMC Pregnancy Childbirth*. 2016;16(1):212. doi:10.1186/s12884-016-1006-9

25. Souza RT, Cecatti JG, Costa ML, et al. Planning, implementing, and running a multicentre preterm birth study with biobank resources in Brazil: The preterm samba study. *Biomed Res Int*. 2019;2019. doi:10.1155/2019/5476350

26. Brasil. Miniistério da Saude. Secretaria de Atenção à Saúde. Departamento de atenção

For peer review only

For peer review only - http://bmjopen.bmj.com/site/about/guidelines.xhtml

Básica. *Orientações Para a Coleta e Análise de Dados Antropométricos Em Serviços de Saúde : Norma Técnica Do Sistema de Vigilância Alimentar e Nutricional - SISVAN / Ministério Da Saúde, Secretaria de Atenção à Saúde, Departamento de Atenção Básica.*; 2011.

27. Fleiss JL. *Statistical Methods for Rates and Proportions*. 2nd ed. New York: John Wiley & Sons, Ltd; 1981.

28. Fletcher RW. *Clinical Epidemiology: The Essentials*. 4th ed. Philadelphia: Lippincott Williams & Wilkins; 2005.

29. Cohen J. Weighted kappa: Nominal scale agreement provision for scaled disagreement or partial credit. *Psychol Bull*. 1968;70(4):213-220. doi:10.1037/h0026256

30. von Elm E, Altman DG, Egger M, Pocock SJ, Gøtzsche PC, Vandenbroucke JP. The strengthening the reporting of observational studies in epidemiology (STROBE) statement: Guidelines for reporting observational studies. *Int J Surg*. 2014;12(12):1495-1499. doi:10.1016/j.ijsu.2014.07.013

31. Fakier A, Petro G, Fawcus S. Mid-upper arm circumference: A surrogate for body mass index in pregnant women. *South African Med J*. 2017;107(7):606-610. doi:10.7196/SAMJ.2017.vi7.12255

32. Bari A, Sultana N, Mehreen S, Sadaqat N, Imran I, Javed R. Patterns of maternal nutritional status based on mid upper arm circumference. *Pakistan J Med Sci*. 2020;36(3):382-386. doi:10.12669/pjms.36.3.1331

33. T.Khadivzadeh. Mid upper arm and calf circumferences as indicators of nutritional status in women of reproductive age. *La Rev Sante Mediterr Orient*. 2002;8(2002):612-618.

34. Benítez Brito N, Suárez Llanos JP, Fuentes Ferrer M, et al. Relationship Between Mid-Upper Arm Circumference and Body Mass Index in Inpatients. *PLoS One*. 2016;11(8):1-10. doi:10.1371/journal.pone.0160480

35. Assefa N, Berhane Y, Worku A. Wealth status, mid upper arm circumference (MUAC) and antenatal care (ANC) are determinants for low birth weight in Kersa, Ethiopia. *PLoS One*. 2012;7(6):e39957. doi:10.1371/journal.pone.0039957

36. Ververs M, Antierens A, Sackl A, Staderini N, Captier V. Which Anthropometric Indicators Identify a Pregnant Woman as Acutely Malnourished and Predict Adverse

For peer review only

For peer review only - http://bmjopen.bmj.com/site/about/guidelines.xhtml

473 Birth Outcomes in the Humanitarian Context? *PLoS Curr*. 2013.
474 doi:10.1371/currents.dis.54a8b618c1bc031ea140e3f2934599c8
475 37. Brasil MS, Secretaria AS, Departamento AB. *Orientações Para a Coleta e Análise de*
476 *Dados Antropométricos Em Serviços de Saúde : Norma Técnica Do Sistema de*
477 *Vigilância Alimentar e Nutricional - SISVAN*. (Ministério da Saúde Departamento de
478 Atenção Básica S de A a S, ed.). Brasília NV - G Estatística e Informação em Saúde:
479 Ministério da Saúde; 2011.
480 38. Mohanty C, Prasad R, Srikanth Reddy A, Ghosh JK, Singh TB, Das BK. Maternal
481 anthropometry as predictors of low birth weight. *J Trop Pediatr*. 2006;52(1):24-29.
482 doi:10.1093/tropej/fmi059
483 39. Van Tonder E, Mace L, Steenkamp L, Tydeman-Edwards R, Gerber K, Friskin D. Mid-
484 upper arm circumference (MUAC) as a feasible tool in detecting adult malnutrition. *South*
485 *African J Clin Nutr*. 2019;32(4):93-98. doi:10.1080/16070658.2018.1484622
486 40. National Department of Health. *Guidelines for Maternity Care in South Africa*. Republic
487 of South Africa; 2015. doi:10.4028/www.scientific.net/AMR.1010-1012.610
488 41. Mahomed K, Williams MA, Woelk GB, et al. Risk factors for pre-eclampsia among
489 Zimbabwean women: maternal arm circumference and other anthropometric measures
490 of obesity. *Paediatr Perinat Epidemiol*. 1998;12(3):253-262. doi:10.1046/j.1365-
491 3016.1998.00116.x
492 42. Ngandu CB, Momberg D, Magan A, Chola L, Norris SA, Said-Mohamed R. The
493 association between household socio-economic status, maternal socio-demographic
494 characteristics and adverse birth and infant growth outcomes in sub-Saharan Africa: A
495 systematic review. *J Dev Orig Health Dis*. 2019:1-18. doi:10.1017/S2040174419000680

496

For peer review only - http://bmjopen.bmj.com/site/about/guidelines.xhtml

Table 1 Anthropometric characteristics of the sample

Characteristics	19-21 (w)	27-29 (w)	1st to 2nd visit p-value*	37-39 (w)	3rd to 1st visit p-value*
**BMI (Kg/m²)**					
Mean±SD	26.33±5.36	27.96±5.36		29.93±5.34	
25th Percentile	22.50	24,20	**<0.0001**	26,30	**<0.0001**
50th Percentile	25.40	27.00		29.00	
75th Percentile	29.20	30.70		32.60	
**Total (n)**	**1,165**	**1,078**		**772**	
**Categories n (%)**					
Underweight	206 (18)	136 (13)		98 (13)	
Adequate	461 (40)	363 (34)	**<0.0001**	275 (36)	**0.0002**
Overweight	299 (26)	392 (36)		219 (28)	
Obese	199 (17)	187 (17)		180 (23)	
**MUAC (cm)**					
Mean±SD	28.71±4.64	29.32±4.54		29.66±4.35	
25th Percentile	25.20	26.00		26.50	
50th Percentile	28.00	28.90	**<0.0001**	29.00	**<0.0001**
75th Percentile	31,50	32,00		32,00	
90% Percentile	35.00	35.50		35.00	
**Total (n)**	**1,139**	**934**		**770**	
**Weight (kg)**					
Mean±SD	67.95±14.69	72.43±14.64		77.65±14.,75	
25th Percentile	57.60	62.33	**<0.0001**	67.50	**<0.0001**
50th Percentile	65.40	70.00		75.10	
75th Percentile	75.70	80.08		85.88	
**Total (n)**	**1,165**	**936**		**772**	

* ANOVA test for repeated measures. Chi square test for BMI categories. From the 3,015 MUAC measures, 8 values were considered a deviation for those seen at the second visit, the mean of the two closest measures recorded was used.

For peer review only - http://bmjopen.bmj.com/site/about/guidelines.xhtml

501 **Table 2.** Diagnostic properties of MUAC screening for maternal nutritional status

Categories	MUAC Cutoff (cm)	Sensitivity % (95% CI)	Specificity % (95% CI)	PPV%	NPV%	Likelihood ratio (LR+)	Likelihood ratio (LR-)	ACC%
**Underweight**								
19-21 (w)	<25.75	88.06 (82.57-92.05)	65.73 (60.76-70.38)	56.91	91.46	3.0	0.14	73.31
27-29 (w)	<25.75	82.09 (74.32-87.97)	77.39 (73.08-81.20)	53.14	93.26	4	0.25	78.51
37-39 (w)	<25.75	77.32 (67.48-84.95)	82.26 (77.44-86.25)	57.69	92.06	3.5	0.38	81.08
**Overweight**								
19-21 (w)	>28.10	72.46 (64.10-79.55)	69.58 (63.58-75.00)	55.56	82.81	2.3	0.43	70.58
27-29 (w)	>28.70	50.21 (43.69-56.73)	84.52 (80.62-87.78)	64.67	75.05	2.5	0.63	72.14
37-39 (w)	>29.45	42.86 (33.36-52.88)	91.16 (87.16-94.03)	63.38	81.71	4	0.67	78.45
**Obese**								
19-21 (w)	>30.15	98.92 (95.78-99.81)	60.39 (55.75-64.85)	50.00	99.29	2.5	0	71.41
27-29 (w)	>30.60	99.44 (96.47-99.97)	70.30 (66.07-74.21)	54.41	99.72	3.3	0	77.95
37-39 (w)	>30.25	94.74 (89.93-97.41)	67.34 (62.45-71.88)	55.48	96.75	3	0.14	75.57

502 Fisher's exact test and Wilson-Brown method which was statistically significant (p<0.05) for all categories at three set points analyzed (p<0.0001). PPV:
503 Positive Predictive Value. NPV: Negative Predictive Value. ACC: Accuracy

For peer review only - http://bmjopen.bmj.com/site/about/guidelines.xhtml

Box 1. Tape color suggested the screening the probability of risk levels for pregnancy complication based on four MUAC cutoffs (cm) for Brazilian pregnant women at three different gestational periods

Color	19-21 (w)	27-29 (w)	37-39 (w)	Interpretation
Red	<25.75 cm			Underweight
Green	25.75 - 28.10	25.75 - 28.70	25.75 - 29.45	Adequate
Yellow	28.11 - 30.15	28.71 - 30.60	29.46 - 30.25	Overweight
Red	>30.15	>30.60	>30.25	Obesity

- Underweight = risk of poor nutrition, indicate support of diet and nutritional counseling
- Adequate = possibly no risk of poor nutrition.
- Overweight = Alert to the need for monitoring weight gain, quality of diet and excessive calories.
- Obesity = risk for adverse outcome of conditions associated with obesity including high blood pressure, gestational diabetes mellitus and large- for-gestational age fetus.

Notes:

Abbreviations: ACC – Accuracy; ANOVA - Analysis of Variance; AUC - Area Under the Curve; BMI - Body Mass Index; CI - Confidence Interval; CNPq - Brazilian National Research Council; CONEP - National Ethics Committee for Research; DRIs - Dietary Reference Intakes; IOM - Institute of Medicine; IRB - Institutional Review Board; MUAC - Mid-Upper Arm Circumference; NCDs - Noncommunicable diseases; NPV - Negative Predictive Value; PPV - Positive Predictive Value; ROC - Receiver Operating Characteristic; SAMBA - Screening And Metabolomics in Brazil and Auckland; SGA - small-for-gestational age; WHO - World Health Organization

Figure 1. Flow chart of women participating in the study

Figure 2. Correlation between BMI and MUAC for each visit (19-21, 27-29, and 37-39 weeks). Dot color indicates the BMI classification. The size of the dots represents maternal weight (kg). Classification: measurements were taken from BMI cutoff values at each week. Rho is the linear correlation coefficient. *Statistical Spearman's correlation test.

19-21(w): 95% confidence interval= 0.8567 to 0.8855, p-value = <0.0001

27-29(w): 95% confidence interval= 0.8532 to 0.8854, p-value = <0.0001

37-39(w): 95% confidence interval= 0.8070 to 0.8522, p-value = <0.0001

For peer review only - http://bmjopen.bmj.com/site/about/guidelines.xhtml

Figure 3. ROC curves for different MUAC cut off values based on BMI categories and the respective Area Under the Curve (AUC) at three different set points during pregnancy

Figure 4. Agreements between MUAC and BMI, according to MUAC categories by elected cutoff values.

In color, the classification is inside boxes (MUAC and BMI). Lines show the MUAC cutoff for each gestational age. The result of the degree of concordance of Cohen's Kappa according to classification at three set points are illustrated below. *Kappa agreement test

19-21(w): Kappa = 0.787, z=24.5, p-value = <0.0001

27-28(w): Kappa = 0.768, z=26.6, p-value = <0.0001

37-38(w): Kappa = 0.765, z=21.5, p-value = <0.0001

For peer review only

For peer review only - http://bmjopen.bmj.com/site/about/guidelines.xhtml

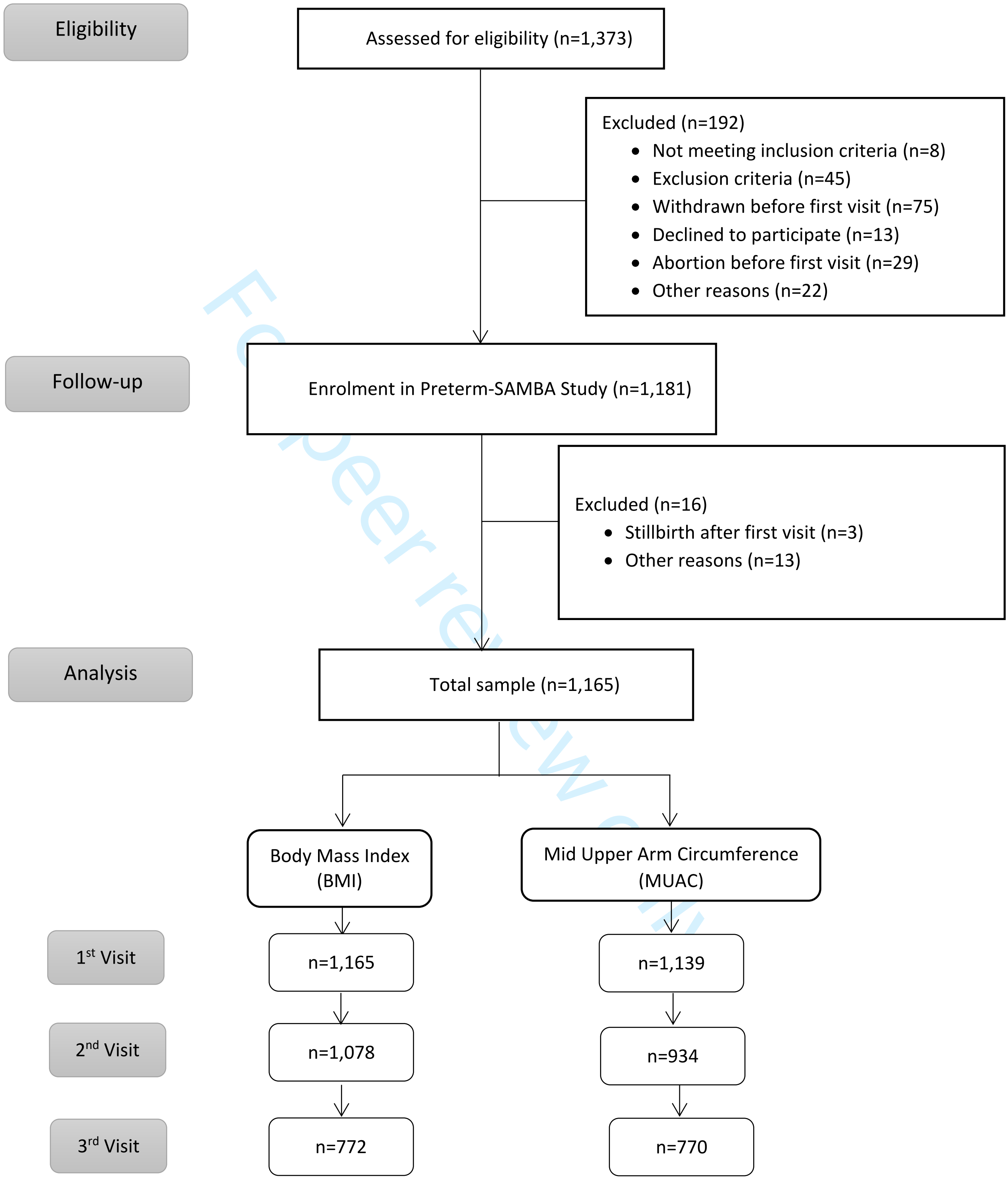
Eligibility
Assessed for eligibility (n=1,373)
Excluded (n=192)
Not meeting inclusion criteria (n=8)
Exclusion criteria (n=45)
Withdrawn before first visit (n=75)
Declined to participate (n=13)
Abortion before first visit (n=29)
Other reasons (n=22)
Follow-up
Enrolment in Preterm-SAMBA Study (n=1,181)
Excluded (n=16)
Stillbirth after first visit (n=3)
Other reasons (n=13)
Analysis
Total sample (n=1,165)
Body Mass Index (BMI)
Mid Upper Arm Circumference (MUAC)
1st Visit
n=1,165
n=1,139
2nd Visit
n=1,078
n=934
3rd Visit
n=772
n=770

19–21(w)

n=1,138

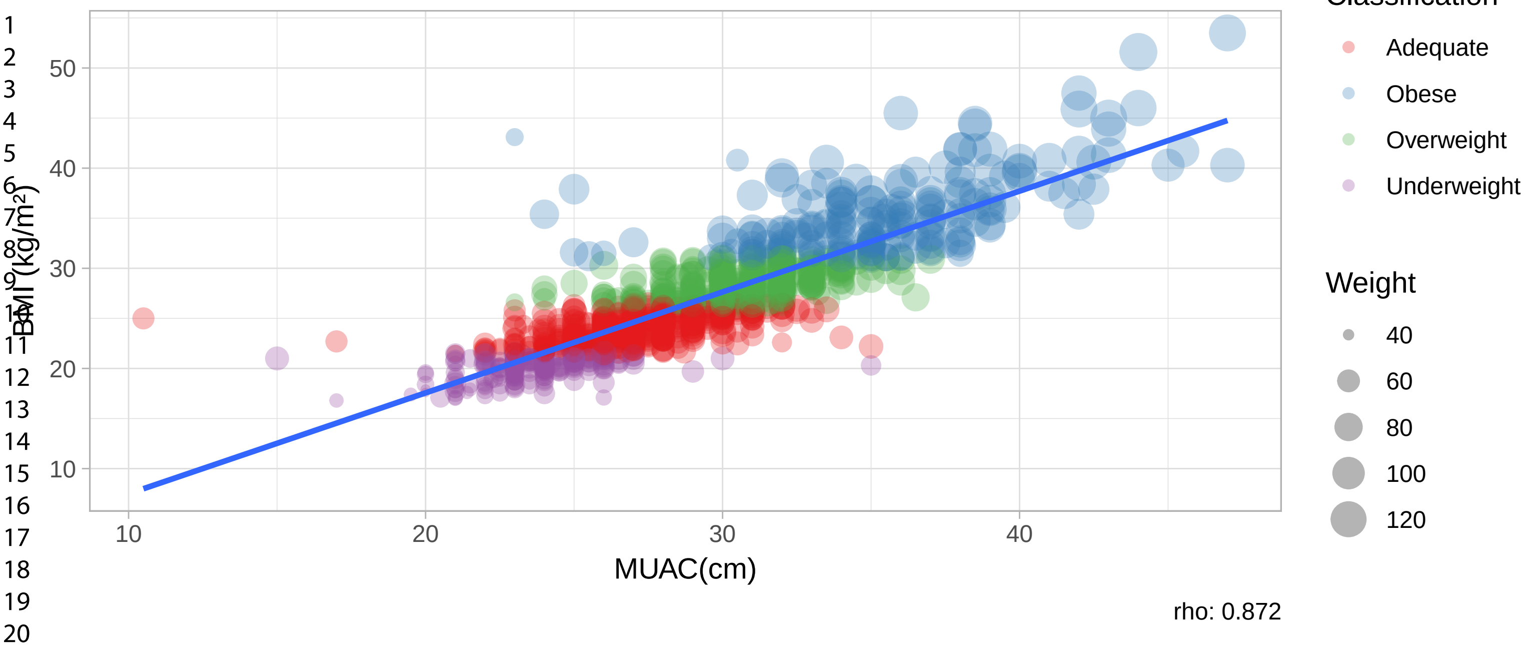

27–29(w)

n=934

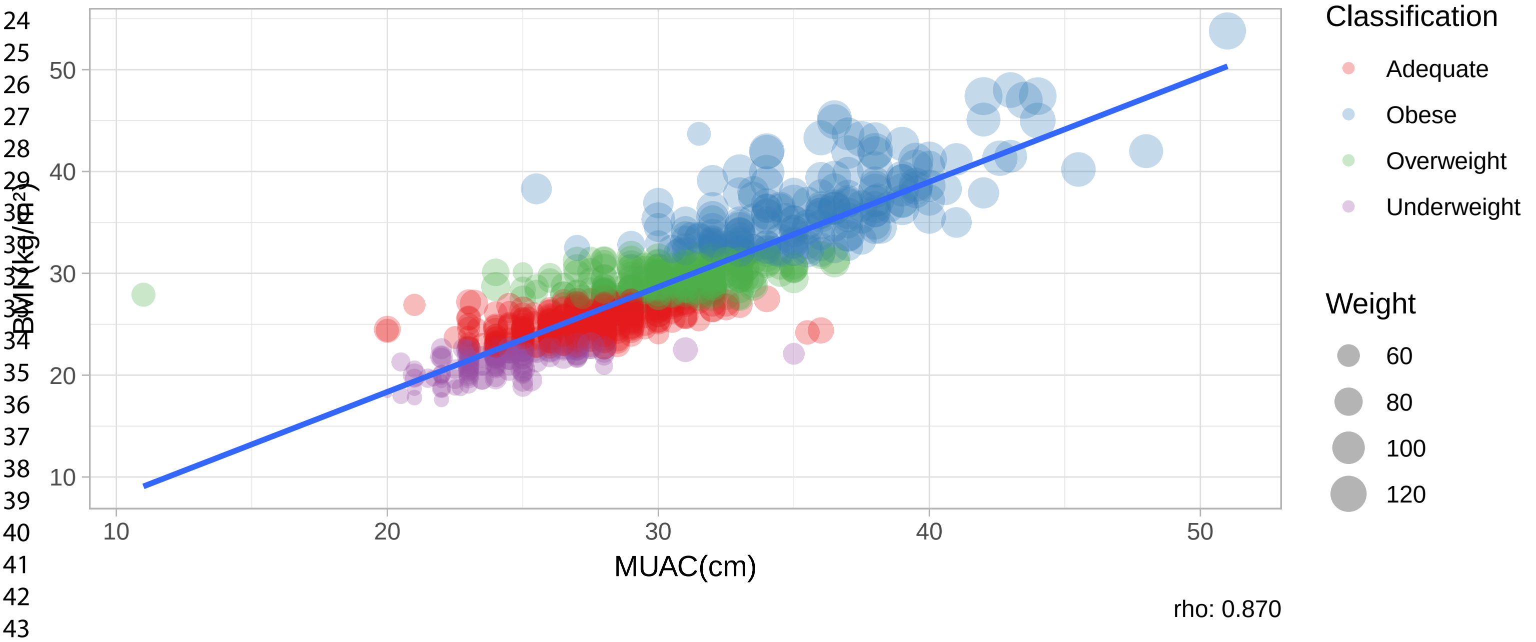

37–39(w)

n=770

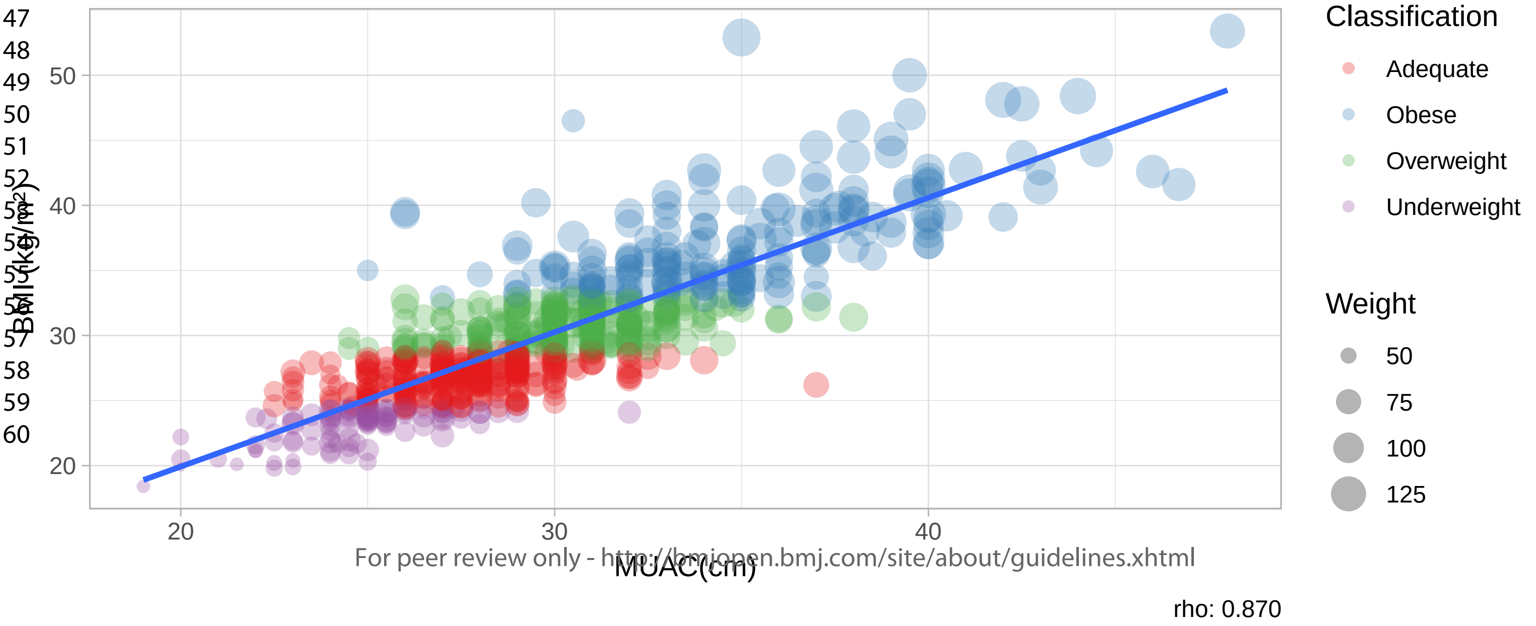

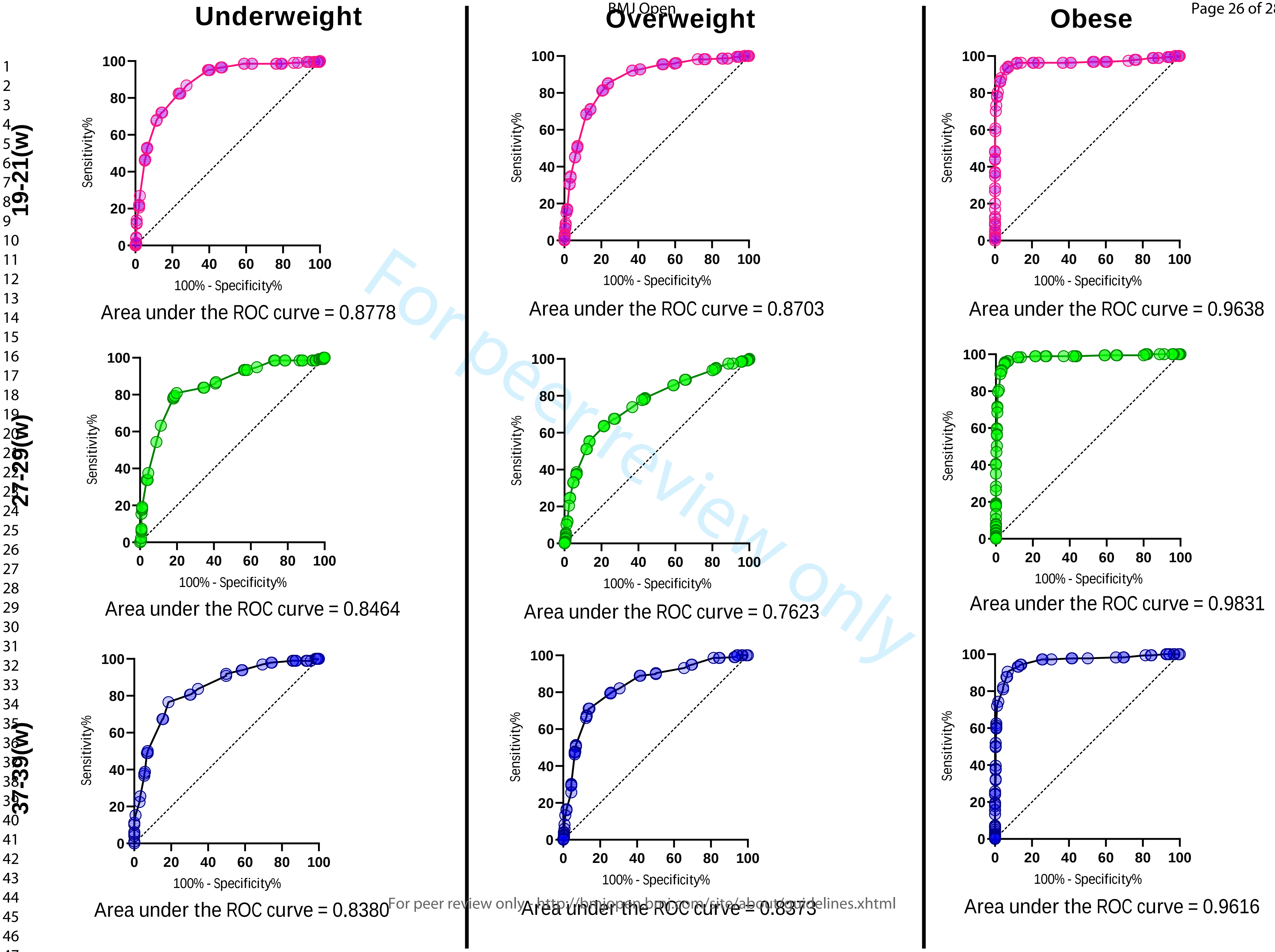

For peer review only

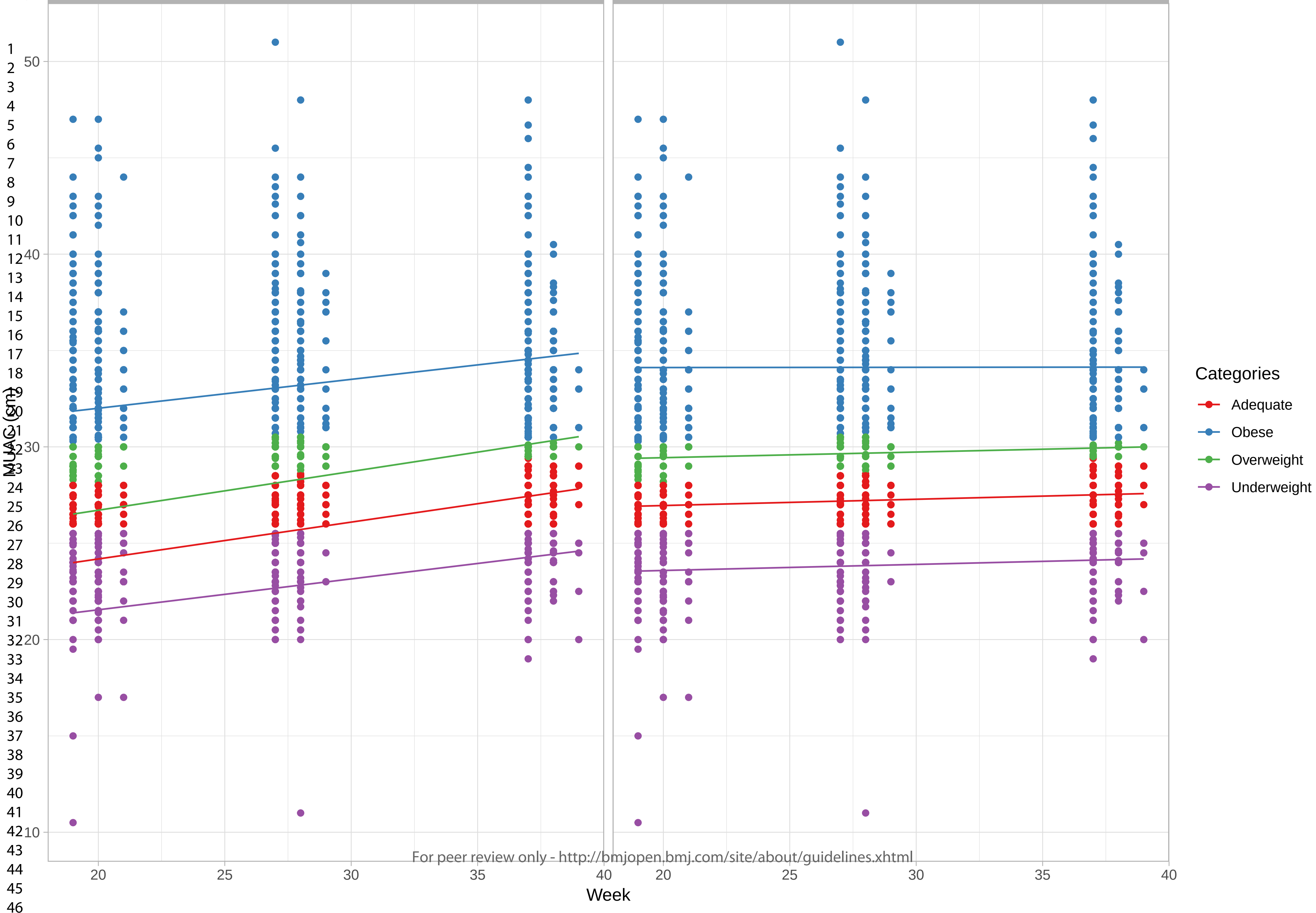

BMI
MUAC
MUAC (cm)
50
40
30
20
10
20
25
30
35
40
Week
Categories
Adequate
Obese
Overweight
Underweight

STROBE Statement—Checklist of items that should be included in reports of ***cohort studies***

	Item No	Recommendation	Page No
**Title and abstract**	1	(*a*) Indicate the study's design with a commonly used term in the title or the abstract	2
		(*b*) Provide in the abstract an informative and balanced summary of what was done and what was found	2
**Introduction**			
Background/rationale	2	Explain the scientific background and rationale for the investigation being reported	3
Objectives	3	State specific objectives, including any prespecified hypotheses	4
**Methods**			
Study design	4	Present key elements of study design early in the paper	4
Setting	5	Describe the setting, locations, and relevant dates, including periods of recruitment, exposure, follow-up, and data collection	4<br>Figure 1
Participants	6	(*a*) Give the eligibility criteria, and the sources and methods of selection of participants. Describe methods of follow-up	4,5
		(*b*) For matched studies, give matching criteria and number of exposed and unexposed	-
Variables	7	Clearly define all outcomes, exposures, predictors, potential confounders, and effect modifiers. Give diagnostic criteria, if applicable	4,5
Data sources/ measurement	8*	For each variable of interest, give sources of data and details of methods of assessment (measurement). Describe comparability of assessment methods if there is more than one group	5
Bias	9	Describe any efforts to address potential sources of bias	6
Study size	10	Explain how the study size was arrived at	4
Quantitative variables	11	Explain how quantitative variables were handled in the analyses. If applicable, describe which groupings were chosen and why	4-6<br>Table 1
Statistical methods	12	(*a*) Describe all statistical methods, including those used to control for confounding	6
		(*b*) Describe any methods used to examine subgroups and interactions	-
		(*c*) Explain how missing data were addressed	4-6
		(*d*) If applicable, explain how loss to follow-up was addressed	5-6
		(*e*) Describe any sensitivity analyses	5-6
**Results**			
Participants	13*	(a) Report numbers of individuals at each stage of study—eg numbers potentially eligible, examined for eligibility, confirmed eligible, included in the study, completing follow-up, and analysed	6-7
		(b) Give reasons for non-participation at each stage	7-8
		(c) Consider use of a flow diagram	Figure 1
Descriptive data	14*	(a) Give characteristics of study participants (eg demographic, clinical, social) and information on exposures and potential confounders	6-7<br>Figure 4
		(b) Indicate number of participants with missing data for each variable of interest	6-8
		(c) Summarise follow-up time (eg, average and total amount)	Table 1
Outcome data	15*	Report numbers of outcome events or summary measures over time	Table 2<br>Figure 3<br>Box 1

For peer review only - http://bmjopen.bmj.com/site/about/guidelines.xhtml

Main results	16	(*a*) Give unadjusted estimates and, if applicable, confounder-adjusted estimates and their precision (eg, 95% confidence interval). Make clear which confounders were adjusted for and why they were included	Table2 Figure2,3
		(*b*) Report category boundaries when continuous variables were categorized	7-8 Figure2,3 Table2 Box1
		(*c*) If relevant, consider translating estimates of relative risk into absolute risk for a meaningful time period	-
Other analyses	17	Report other analyses done—eg analyses of subgroups and interactions, and sensitivity analyses	Figure2,3 Table 2
**Discussion**			
Key results	18	Summarise key results with reference to study objectives	8
Limitations	19	Discuss limitations of the study, taking into account sources of potential bias or imprecision. Discuss both direction and magnitude of any potential bias	11,12
Interpretation	20	Give a cautious overall interpretation of results considering objectives, limitations, multiplicity of analyses, results from similar studies, and other relevant evidence	8-11
Generalisability	21	Discuss the generalisability (external validity) of the study results	11,12,13
**Other information**			
Funding	22	Give the source of funding and the role of the funders for the present study and, if applicable, for the original study on which the present article is based	12

*Give information separately for exposed and unexposed groups.

Note: An Explanation and Elaboration article discusses each checklist item and gives methodological background and published examples of transparent reporting. The STROBE checklist is best used in conjunction with this article (freely available on the Web sites of PLoS Medicine at http://www.plosmedicine.org/, Annals of Internal Medicine at http://www.annals.org/, and Epidemiology at http://www.epidem.com/). Information on the STROBE Initiative is available at http://www.strobe-statement.org.

For peer review only

For peer review only - http://bmjopen.bmj.com/site/about/guidelines.xhtml

BMJ Open

The proposal of MUAC as a fast tool to monitor pregnancy nutritional status: results from a cohort study in Brazil

Journal:	*BMJ Open*
Manuscript ID	bmjopen-2020-047463.R1
Article Type:	Original research
Date Submitted by the Author:	19-Apr-2021
Complete List of Authors:	Miele, Maria; State University of Campinas Faculty of Medical Sciences, Obstetrics and Gynecology<br>Souza, Renato; Universidade Estadual de Campinas, Obstetrics and Gynecology<br>Calderon, IMP; UNESP Campus de Botucatu, Department of Gynecology and Obstetrics<br>Feitosa, Francisco; Federal University of Ceara, Obstetric Department of MEAC<br>Leite, Debora; Federal University of Pernambuco, Obstetrics and Gynecology<br>Rocha Filho, Edilberto; Federal University of Pernambuco, Obstetrics and Gynecology<br>Vettorazzi, Janete; Federal University of Rio Grande do Sul, Obstetrics and Gynecology<br>Mayrink, Jussara; Universidade Estadual de Campinas Faculdade de Ciencias Medicas, Obstetrics and Gynecology<br>Fernandes, Karayna; State University of Campinas Faculty of Medical Sciences, Obstetrics and Gynecology<br>Vieira, Matias; King's College London School of Life Course Sciences, Division of Women's Health<br>Pacagnella, Rodolfo; State University of Campinas, Obstetrics and Gynecology<br>Cecatti, Jose; State University of Campinas Faculty of Medical Sciences, Obstetrics and Gynecology
**Primary Subject Heading**:	Obstetrics and gynaecology
Secondary Subject Heading:	Nutrition and metabolism
Keywords:	Maternal medicine < OBSTETRICS, NUTRITION & DIETETICS, Prenatal diagnosis < OBSTETRICS

SCHOLARONE™
Manuscripts

I, the Submitting Author has the right to grant and does grant on behalf of all authors of the Work (as defined in the below author licence), an exclusive licence and/or a non-exclusive licence for contributions from authors who are: i) UK Crown employees; ii) where BMJ has agreed a CC-BY licence shall apply, and/or iii) in accordance with the terms applicable for US Federal Government officers or employees acting as part of their official duties; on a worldwide, perpetual, irrevocable, royalty-free basis to BMJ Publishing Group Ltd ("BMJ") its licensees and where the relevant Journal is co-owned by BMJ to the co-owners of the Journal, to publish the Work in this journal and any other BMJ products and to exploit all rights, as set out in our licence.

The Submitting Author accepts and understands that any supply made under these terms is made by BMJ to the Submitting Author unless you are acting as an employee on behalf of your employer or a postgraduate student of an affiliated institution which is paying any applicable article publishing charge ("APC") for Open Access articles. Where the Submitting Author wishes to make the Work available on an Open Access basis (and intends to pay the relevant APC), the terms of reuse of such Open Access shall be governed by a Creative Commons licence – details of these licences and which Creative Commons licence will apply to this Work are set out in our licence referred to above.

Other than as permitted in any relevant BMJ Author's Self Archiving Policies, I confirm this Work has not been accepted for publication elsewhere, is not being considered for publication elsewhere and does not duplicate material already published. I confirm all authors consent to publication of this Work and authorise the granting of this licence.

For peer review only

ORIGINAL RESEARCH - Miele.M.J.O et al

The proposal of MUAC as a fast tool to monitor pregnancy nutritional status: results from a cohort study in Brazil

Maria J O Miele[1]
Renato T Souza[1]
Iracema M P Calderon[2]
Francisco E Feitosa[3]
Débora F Leite[1,4]
Edilberto A Rocha Filho[4]
Janete Vettorazzi[5]
Jussara Mayrink[1]
Karayna G Fernandes[1]
Matias C Vieira[1,6]
Rodolfo C Pacagnella[1]
José G. Cecatti[1]
For the Preterm SAMBA study group*

[1] Department of Obstetrics and Gynecology, University of Campinas (UNICAMP), School of Medicine, Campinas, SP, Brazil; [2] Department of Gynecology and Obstetrics, Botucatu Medical School, Sao Paulo State University (Unesp), Botucatu, SP, Brazil;[3] Obstetric Department of MEAC - Maternity School of the Federal University of Ceará, Fortaleza, CE, Brazil; [4] Department of Maternal and Child Health, Federal University of Pernambuco, Recife, PE, Brazil; [5] Department of Obstetrics and Gynecology, Maternity Hospital, Federal University of RS, Porto Alegre, RS, Brazil; [6] Division of Women and Children's Health, School of Life Course Sciences, Faculty of Life Sciences and Medicine, London, United Kingdom.

*The other members of the Preterm SAMBA study group are list in the Acknowledgements session

Correspondence: José Guilherme Cecatti
Department of Obstetrics and Gynecology, State University of Campinas
R. Alexander Fleming, 101 - Cidade Universitária, Zip Code 13081-970 - Campinas, SP – Brazil
Tel 55.19.35219482
Fax 55.19.35219304
Email : cecatti@unicamp.br

For peer review only

Abstract:

Objective: In Brazil, although the assessment of maternal nutritional status is recommended using Body Mass Index (BMI), this is only possible in settings adequately prepared. Mid-Upper Arm Circumference (MUAC) is another biological variable identified as a tool for rapid assessment of nutritional status that is correlated with BMI. Therefore, we aim to surrogate BMI by MUAC cut-offs for rapid screening of maternal nutritional status starting at mid-pregnancy**.**

Design: analysis of the multicenter cohort study entitled "Preterm SAMBA" using an approach of validation of diagnostic test,

Setting: outpatient prenatal care clinics from five tertiary maternity hospitals from three different Brazilian regions.

Participants: 1,165 pregnant women attending prenatal care services from 2015 to 2018 and with diverse ethnic characteristics who were enrolled at mid-pregnancy and followed in three visits at different gestational weeks.

Primary and secondary outcome measures: sensitivity, specificity, positive and negative predictive values, likelihood ratio and accuracy of MUAC being used instead BMI for the assessment of nutritional status of women during pregnancy.

Results: We found a strong correlation between MUAC and BMI, in the three set points analyzed (rho: 0.872, 0.870, and 0.831, respectively). Based on BMI categories of nutritional status, we estimated the best MUAC cut-off points, finding measures according to each category: underweight <25.75cm (19-39 week); overweight 28.11 - 30.15cm (19-21 week), 28.71 - 30.60cm (27-29 week), and 29.46 - 30.25cm (37-39 week); and obese >30.15cm (19-21 week), >30.60cm (27-29 week) and >30.25cm (37-39 week) per gestational week. Therefore, we defined as adequate between 25.75 - 28.10cm (19-21 week), 25.75 - 28.70cm (27-29 week), and 25.75 - 29.45cm (37-39 week) of MUAC.

Conclusion: We conclude that MUAC can be useful as a surrogate for BMI as a faster screening of nutritional status in pregnant women.

For peer review only

For peer review only - http://bmjopen.bmj.com/site/about/guidelines.xhtml

Keywords: Body Composition. Mid-upper arm circumference; Body Mass Index; nutritional status; Prenatal Nutritional Risk Factors; Pregnancy Outcome.

Strengths and limitations of this study

- This study was a multicenter cohort performed in different regions of the country and exploring changes in maternal body composition.
- The assessment was standardized carried out by maternity health teams and included in the system in real time.
- Although the sample size is robust, the original study was not specifically designed for this assessment, being an analysis of secondary objectives

Introduction

Antenatal care represents a window of opportunity to promote healthy life habits, provide individualized care and prevent adverse health outcomes [1]. Worldwide, the concern about obesity can be extended to maternal underweight and malnutrition, with short and long-term consequences [2]. The Institute of Medicine (IOM) developed a guideline for American pregnant women to monitor maternal weight gain based on the World Health Organization (WHO) body mass index classification (BMI)[3]. Public health system in Brazil is monitoring the pregnancy nutritional status by the weight gain, when information on pre-pregnancy weight is available, or by the BMI charts of Atalah according to gestational week. [4]. However, it is necessary to measure maternal weight and height early in pregnancy. In the absence of this information, self-reported weight should be used [5]. In low or middle-income countries, it is common for women to seek antenatal care very late. It is also not uncommon for women to skip antenatal care altogether, resulting in missing information on weight before or during pregnancy. As a result, monitoring of

For peer review only - http://bmjopen.bmj.com/site/about/guidelines.xhtml

nutritional status by BMI becomes less effective [6–8]. Moreover, there is a tendency to underestimate weight and overestimate height when measures are dependent on maternal recall. Anthropometric measurements are frequently not recorded in the prenatal booklet, compromising the accuracy of nutritional status assessment [9–11].

Mid-Upper Arm Circumference (MUAC) has been recognized as a fast tool adopted to monitor nutritional status, and it is strongly correlated with BMI [12]. It has already been implemented for adult care in under-resourced settings, and for monitoring maternal undernourishment and fetal growth [13–15].Thus, MUAC allows the assessment of protein intake and storage, related to severe undernutrition [16]. Despite the global obesity pandemic coexisting with undernutrition, there is a maternal and fetal health risk of developing non-communicable diseases such as hypertension, diabetes, and delivery of preterm small-for-gestational age (SGA) neonates, compromising the health of the future offspring [17,18]. Poverty and malnutrition are strongly associated with low birthweight and stunted growth in the first 1000 days of life [18–21]. Brazil has a big variation on demographic density, with higher densities in the South/Southeast regions and Northeast coastal compared to the Northern and Central-West regions with larges empty spaces occupied by the Amazon rainforest and turned to agriculture and livestock[22,23]. In this country with socioeconomic disparities, we have an opportunity to investigate in a representative sample an option to faster screening risk of maternal nutritional status from mid-pregnancy onwards, allowing risk assessment related to abnormal nutrition, which may affects pregnancy outcomes. Implementing an easy and reproducible tool to monitor nutritional status is a great opportunity for scaling-up risk assessment related to abnormal and suboptimal nutritional conditions, monitor pregnancy nutritional interventions, and facilitate the provision of a more equitable antenatal care.

Material and methods

Study design

This study corresponds to a secondary objective of the multicenter cohort study entitled "Preterm SAMBA - Preterm Screening and Metabolomics in Brazil and Auckland," including 1,165 Brazilian nulliparous women with singletons, and no history of previous severe clinical condition. Detailed

For peer review only - http://bmjopen.bmj.com/site/about/guidelines.xhtml

106 information on research methods and procedures of this study has already been fully explored and published elsewhere [24,25]. Data were collected between 2015 and 2018 at five Brazilian hospitals located in three different geographical regions that have distinctive physical and ethnic characteristics (Maternity Hospital of the University of Campinas (CAISM) and Maternity Hospital from Botucatu Medical School in the Southeast; Maternity of the Hospital of Clinics, Federal University of Rio Grande do Sul in the South; and Clinics Hospital, Federal University of Pernambuco and Maternity School Assis Chateaubriand of the Federal University of Ceará in the Northeast). Pregnant women receiving routine prenatal care who met the inclusion criteria were included in the study between 19 and 21 weeks of gestation for the first study interview, in which data collection and anthropometric measurements were recorded (Figure 1).

Study procedures

The coordinating center for this study was the University of Campinas (CAISM) where the first research meeting took place with health professionals from each center. These professionals were trained by a nutritionist to take anthropometric measurements in pregnant women. Anthropometric measurements were obtained at three set points: 19-21, 27-29 and 37-39 weeks. We excluded from the analysis data from women with stillbirth, preterm birth, and from those considered as loss of follow up. All centers inserted maternal anthropometry data into the database of the study (MedSciNet® AB, Sweden). Height (cm) and weight (kg) were measured once in each of the three scheduled study visits by a stadiometer and weighing scale, with the feet close to the heels, buttocks, and shoulder blades aligned and the head positioned in the horizontal plane of Frankfurt. From the results of height and weight, an automatic estimate of Body Mass Index (BMI) was generated by software using the formula: weight/height^2. The Mid-Upper Arm Circumference (MUAC) was measured with a flexible non-extensible tape. The left arm circumference was measured in centimeters at the mid-point between the acromion and shoulder by trained health professionals using a standard scale. All MUAC anthropometric measurements were performed three times and the mean was recorded. Some cases from the early beginning of the study had no MUAC measurements while the team was not enough trained to measure it. Two researchers checked all MUAC results. When the variation exceeded the average of the values of the three measures, the mean of the two closest recorded measures

For peer review only - http://bmjopen.bmj.com/site/about/guidelines.xhtml

was used.. BMI values were categorized by the Atalah curve[4], based on the Brazilian Ministry of Health´s reference to monitor pregnancy[26], identify adult maternal nutritional status and develop MUAC cut-off points.. According to this criterion, the Atalah's classification for "Adequacy" was used as lower risk for malnutrition and the other categories were considered more likely to develop malnutrition. The adolescents of this sample were 13% and, considering that there is not a specific tool for assess this group[27], all women were analyzed by the Atalah´s charts. This instrument was used to identify nutritional status in pregnant women according to BMI and develop MUAC cut-off points. Sociodemographic characteristics, including maternal age, skin color and family income were self-reported and included into electronic platform of the study.

Statistical analysis

To compare BMI, MUAC, and maternal weight measurements and investigate whether differences in values exist for the measures, a repeated measures one-way ANOVA test was used. Statistical significance was determined using the Bonferroni's multiple comparisons test, with alpha = 0.05. Comparisons were made between three set points (at different gestational ages). For categorical variables, chi-square tests were performed to analyze differences between BMI classifications at each set point. Each row was analyzed individually, without assuming a consistent SD. Then, a post hoc test was run to assess the differences between all values and find the result of each comparison, using Dunn's multiple comparisons test. Histograms of maternal MUAC and BMI were constructed to determine the distribution of each measurement for each set point separately and for all datasets combined. The Normal QQ plot Normality and Log normality tests were applied using the Shapiro-Wilk test and the Kolmogorov-Smirnov test to compare normal and lognormal distributions. Spearman's correlation coefficient was applied to estimate the linear relationship between MUAC and BMI. Afterwards, the Receiver Operating Characteristic (ROC) curve was plotted to obtain the performance of MUAC measures to predict maternal BMI for all possible cut-off points. The area under the curve (AUC) was calculated along with its 95% confidence interval (CI). Based on coordinates of the curve, the most accurate MUAC cut-off point was selected for the MUAC variable. For each BMI cutoff, we calculated a 2x2 table showing the cross-tabulation of MUAC measurement and calculated accuracy using McNemar's test of concordance (continuity corrected) [28]. To estimate sensitivity, specificity, Negative

For peer review only - http://bmjopen.bmj.com/site/about/guidelines.xhtml

Predictive Value (NPV), and Positive Predictive Value (PPV), BMI was calculated for each gestational week considering normal the results from BMI adequacy classification. From this categorization, MUAC values were selected according to BMI references. The Youden method was calculated (highest sensitivity + specificity -1) to identify the best cut-off measures based on the largest vertical distance between the ROC curve and the diagonal curve, for each visit and separated for classification. The positive and negative Likelihood Ratio values was calculated according to Fletcher [29]. For the agreement between BMI and MUAC measurements, weighted Cohen's Kappa coefficient was used. Ordinal responses with disagreements were calculated according to their squared distance from the perfect agreement (squared weights)[30].

Ethics statement

The current study was approved by each local Institutional Review Board (IRB) and amended by the Brazilian National Committee for Ethics in Research (CONEP) - Letter of approval 1.048.565 issued on 28th April 2015. The study complies with national and international regulations for human being experiments, including resolution CNS 466/12 of the Brazilian National Heath Council and the 1989 Declaration of Helsinki. All women signed an informed consent form before enrolment. This study follows the Declaration of Strengthening Report on Observational Studies in Epidemiology[31].

Patient and public involvement

Patients or the public were not involved in the design, or conduct, or reporting, or dissemination plans of our research

Results

Out of a total of 1,165 nulliparous women (Figure 1), 461 (39.6%) were from the Southeast, 139 (11.9%) from the South and 565 (48.5%) from the Northeast of Brazil. Maternal age at the first visit corresponded to 291 (25.0%) women younger than 20 years (97 under age 17 years), 796 (68.3%) aged 20 to 34 years, and 78 (6.7%) older than 34 years. From the total sample, 578 (49.6%) women self-declared as having brown skin, 117 (10.0%) as having black skin, 462

For peer review only - http://bmjopen.bmj.com/site/about/guidelines.xhtml

(39.7%) as white skin and 7 (0.6%) as yellow, and 1 (0.1%) as having another ethnicity. Of this sample, 52 (4.5%) women lived with less than U$ 250 per month, 253 (21.7%) received from U$ 251 to 500; 380 (32.6%) women had a monthly income ranging from U$ 501 to 1,000 and 480 had an income over U$: 1,000 (41.2%).

Table 1 shows the distribution of anthropometric measurements, including the BMI of all participating women according to each set point. The mean variation in BMI measurements throughout pregnancy was greater than MUAC, and the opposite was observed in extreme measures (minimum and maximum), with greater variations in MUAC and lower BMI.

Figure 2 shows the graphic correlation between BMI and MUAC according to each pregnancy set points measured at the prenatal visits. .. BMI categories were represented by four different colors and dot size differentiated maternal weight.

To determine the most accurate MUAC value, different categories of nutritional status were selected by BMI classification obtained at three standard prenatal visits scheduled. Differences were significant ($p<0.05$) for all categories and for three set points analyzed ($p<0.0001$). The Area Under the Curve (AUC) was statistically positive at three set points, and robustly significant for screening extreme nutritional status, notably for obese women (Figure 3).

The AUC showed that MUAC had a high discrimination capacity for nutritional status obtained by the actual gold standard method (BMI). Table 2 shows MUAC cutoff measures analyzed for use as a screening method for maternal nutritional status. At the first prenatal visit, accuracy was lower than at other gestational ages assessed. It was noteworthy, however, that a substantial difference existed between the predictive values, although the calculus of Likelihood (positive and negative) confirmed a slight probability of screening women at risk for malnutrition (obese, underweight, or overweight). There was no probability of screening for the lack of risk. The diagnostic test indicated that this measure was a good alternative to the screening test, especially for obese and underweight women. Measures between the cutoff for underweight and overweight were identified as within normal, or desirable. Afterwards, an interaction charter between MUAC and BMI classifications was constructed, using selected MUAC measures as a model for gestational age. It is possible to observe differences among the curves, with a better agreement for extremes of classification (obese and underweight). Figure 4 shows changes after the second set point or from the 27th week of gestation onwards, with a better relationship

For peer review only

For peer review only - http://bmjopen.bmj.com/site/about/guidelines.xhtml

between categories. Comparing the concordance between categories of the two different methods of assessment, a substantial agreement was shown reporting an excellent agreement beyond chance (Figure 4). Box 1 presents as a result of our proposal to monitor the maternal nutritional status by cut-offs for the MUAC tape with colors according to the classifications and degrees of risk.

Discussion

This study investigated the hypothesis that the MUAC cutoff, at different stages of pregnancy, should be a feasible alternative to BMI and is a quicker tool for screening maternal nutritional status in Brazilian pregnant women. We confirmed a strong correlation between BMI and MUAC of Brazilian pregnant women at three set points covering distinct gestational weeks: mid pregnancy (19-21w), early third trimester (27-29w) and late pregnancy (37-39w). We achieved our goals of identifying MUAC cut-off values for different gestational weeks and malnutrition risk levels and present a simple tool as an option to faster screening maternal nutritional status from mid-pregnancy to 39 weeks.

Our results are similar to findings obtained by studies that have tested this correlation at early and late gestational age, with diverse sociodemographic characteristics [6,32,33]. Most studies have associated MUAC with maternal malnutrition linked to underweight, obesity, and low birthweight (LBW). In addition, some authors have assessed MUAC in non-pregnant women and correlated those cutoffs with adult BMI in their own population [8,34–36].

Effects of inadequate nutritional status during pregnancy increases the risk for low birthweight, preterm birth[37], and small or large for gestational age, among others[38]. Therefore, MUAC has been used as an anthropometric measurement in routine antenatal care to detect malnutrition focused on low maternal weight in middle and low-income countries [39]. We identified that a single MUAC measure of (<25.75cm) is a predictor of underweight and poor nutritional risk. However, a literature review addressing which anthropometric values indicative of acute malnutrition were associated with adverse birth outcomes, proposed a cutoff point of 23cm to identify undernourished women. This value was considered to be correlated with lower pre-pregnancy BMI measures (<18.5kg/m^2) and the occurrence of adverse pregnancy outcomes. Our thresholds are in accordance with the Brazilian recommendation for BMI at mid-pregnancy. The

For peer review only - http://bmjopen.bmj.com/site/about/guidelines.xhtml

reference value of the Health System for Brazilian pregnant women at 6 weeks of gestation is a BMI <19.9kg/m^2, close to the IOM recommendations of a BMI of <19.8 kg/m^2 [3,40].

Although MUAC is considered a stable measure, with less variation in comparison to BMI [17], a slight increase on MUAC curves was clearly observed throughout pregnancy. There was a smooth MUAC curve contrasting to the increasing sharp trace of the BMI curve, with a significant agreement among assessments. Our results show not only changes in measures associated with poor nutritional status, but also a mild change in other classifications. A prior study with women in Argentina tested MUAC cut-off points using the curves generated in their second trimester of gestation to detect LBW. The authors indicated a slight difference in MUAC cut-offs for undernutrition status according to gestational week, as well as a MUAC of 24.5cm for pregnant women ≤16 weeks, <25.5cm at 28 weeks and <26.5cm at 36 weeks [6]. In India, MUAC was assessed in the first trimester to detect LBW, with an accuracy of 58.7%, as well as positive and negative predictive values of 38.8% and 76.8%, respectively. The authors defined <22.5cm as the best MUAC cutoff that alerts to an underweight measure and is a predictor of LBW in the first trimester [41]. Our AUC values indicate a good diagnostic accuracy of this test between BMI and MUAC. The results showed a high degree of accuracy of MUAC cutoffs satisfactorily beating the current standard of classification for nutritional status (Table 2). In terms of correlations between values of MUAC, those results from India are similar to ours, showing a better capacity to find more women who are not at risk, but not missing any women at risk for malnutrition. Nevertheless, in the absence of a 'gold standard' for nutritional risk screening, an African study using a combination of validity tools to diagnose malnutrition found a significant association between MUAC, BMI and the Malnutrition Universal Screening Tool (MUST), searching for poor nutritional status in the adult population (men and women). However, BMI and MUST missed undernutrition in 38% and 43% patients, respectively, whereas MUAC was tracked in 100%. In South Africa, a MUAC value <23cm was adopted for severely underweight (BMI < 16 kg/m^2). A value of < 24 cm (BMI < 18.5 kg/m^2) was used to screen for malnutrition or a population at risk for malnutrition [42].

In our study, a MUAC value of 25.75 was used to identify low maternal weight (19 - 21 weeks), different from the value proposed in the literature, referring to early gestation or a different population. Although there was a better agreement among values between <22 and <23cm to identify LBW, a MUAC of <23cm is more highly recommended for the African and Asian contexts,

For peer review only - http://bmjopen.bmj.com/site/about/guidelines.xhtml

noting that these values were not linked to gestational age [39]. The Khadivzadeh study with healthy women of reproductive age, indicated that a value <24cm was the best MUAC measure to detect underweight Iranian women [34]. A study in Spain with 1,373 male and female subjects of different ages indicated that a MUAC ≤22.5cm correlates more closely with BMI <18kg/m^2 [35].

The Guidelines for Maternity Care in South Africa monitor maternal nutritional status in antenatal screening using MUAC measures of <23cm, categorized as underweight. For the group of underweight women, this requires a search for infection and neoplasia. Fetal growth assessment and nutritional support are necessary [43]. A study on the effect of obesity, diet and preeclampsia conducted in Ethiopia adopt MUAC classification values <23cm for underweight, and between 23 and <25cm for adequate. Those authors observed that women with a MUAC <25cm had healthier habits and ate more vegetables and fruits. Along with the group of MUAC 21-23cm, those women have a three-fold lower chance of developing preeclampsia. Women with MUAC between 23–24.99cm were considered to have optimal status and <23cm was categorized as underweight [17]. Similarly, our first cut off measure that is likely to determine diseases linked to overweight/obesity such as preeclampsia, starts at a MUAC of 28.11cm. It is important to highlight the difficulty in screening for disorders in pregnant women in the first trimester of pregnancy in some countries. The rate of the first antenatal care visit in the first trimester of pregnancy was estimated to be 58·6% worldwide, whereas it was 48·1% in developing countries and 24·0% in low-income countries[7].

For the conditions associated with excessive weight (overweight and obesity), South African Guides to maternal care classify a MUAC ≥30cm as obese. This measurement raises concern for hypertension, preeclampsia and gestational diabetes, guiding actions to prevent fetal macrosomia and complications during labor and delivery [43]. In Ethiopia, a MUAC ≥25cm is categorized as overweight and obese, where authors highlight that MUAC measurements between 28 and 39cm are more capable of predicting complications such as preeclampsia [17]. These parameters originated from a previous study in Zimbabwe with a black African population. Those authors found that women with a MUAC between 28 and 29 cm had a fourfold higher risk than those who had a MUAC between 21 and 23 cm [44].

From a sample of 2000 women, ranging from 15-45 years of age in the Islamic Republic of Iran with a BMI >29kg/m^2, it was determined that a MUAC measure >30.5cm can estimate BMI,

For peer review only

detect nutritional disorders, and is useful to search for obesity [34]. Previously, a meta-analysis using data from individual participants in seven countries explored the potential to find a significant MUAC cutoff point to identify malnutrition in pregnant women. Mean MUAC varied widely between countries evaluated, as well as according to the weeks in which measurements were taken. In early pregnancy, measurements were between 21.8 and 23.0 cm. In the second and third trimester of pregnancy, averages were 25.2 and 26.5 cm. In the postpartum period, the average ranged from 27.1 to 28.9 cm. The authors concluded that MUAC cut-off values can vary widely internationally and were dependent on the population and time of assessment [12].

There seems to be a wide difference in MUAC according to the population, age of pregnant women, and gestational week for the underweight category (21 to 24cm), and particularly in the search for the overweight/obesity category (29.0cm to >33cm). We presumed that nutritional status was adequate at measurements between >24cm and <29cm. MUAC cut-off values found in the literature were commonly comparable to pre-pregnant BMI, with an extremely small difference between those values and our results at mid-pregnancy. The consequences of extreme anthropometric measurements are linked to adverse outcomes for the mother and child. The socioeconomic, educational and ethnic diversity of the Brazilian population is tremendous, and these characteristics can influence maternal body composition [45].

MUAC must be specific to different populations. The originality of the study was to investigate a cohort of over a thousand pregnant women from different regions of Brazil. In the Brazilian pregnant population, there are no studies showing that MUAC is effective at screening risks for extreme cut-offs measures (low weight and overweight/obesity).

We assessed a tool that, to be applied, uses only a tape and can be adopted by obstetricians in the prenatal routines, which is capable of alerting to the risks of inadequate nutrition and their probable consequences linked by the levels of malnutrition, especially needed in settings were anthropometric measures are limited or missing. Once any measure indicating inadequacy is tracked, the need for investigation and eventual support of the diet and nutritional counseling, in addition to monitoring blood pressure and blood glucose, is highlighted.

A possible limitation is that this analysis was conducted as a secondary objective of the original study that had no specific sample size calculation. Therefore, the number of women included could be insufficient for all the associations assessed. All the maternities involved in this

For peer review only - http://bmjopen.bmj.com/site/about/guidelines.xhtml

study are referral hospitals receiving women from nearby small towns, and therefore, we had a higher rate of loss to follow up of the prenatal visits with their corresponding measurements. Strengths include prior training of health professionals, real-time data inclusion, use of additional statistical procedures to explore results and mainly, a MUAC cutoff point defined for a Brazilian population of pregnant women (at three regions of the country, with different regional characteristics).

.

Conclusion

In conclusion, MUAC has the advantages over BMI due to simplicity of application. It does not require the calculation of other measures and is independent of pre-pregnancy weight recall. In a multiethnic population, our MUAC cut-off measures could be used as a faster screening tool of nutritional status in pregnant women. It may track risk and provide timely intervention to avoid adverse pregnancy outcomes. Still, more research will be welcome in order to validate these cutoffs for pregnancy outcomes.

Acknowledgments

The Preterm SAMBA study group also included: Maria L Costa, Mary A. Parpinelli, Rafael B Galvão, José Paulo Guida, Danielly S Santana, Bianca F. Cassettari, Daisy de Lucena, Denise F. Cordeiro, Elias F Melo Junior, Danilo Anacleto, Lucia Pfitscher, Luiza Brust.

This study was granted jointly by the Brazilian National Research Council (CNPq) (Award 401636/2013-5) and the Bill and Melinda Gates Foundation (grant OPP1107597). The funders played no role whatsoever in study design, writing of the manuscript or in the decision to submit the manuscript for publication.

Disclosure

The author reports no conflicts of interest in this work.

For peer review only - http://bmjopen.bmj.com/site/about/guidelines.xhtml

Data availability

Data are available upon reasonable request

Funding

This study was selected for sponsoring from the research call “Grand Challenges Brazil: Reducing the burden of preterm birth” number 05/2013 jointly issued by the Brazilian National Research Council (CNPq) (Award 401636/2013-5) and the Bill and Melinda Gates Foundation (grant OPP1107597). The funders played no role whatsoever in the study design, writing of the manuscript nor in the decision to submit the manuscript for publication.

For peer review only

Authors contributions

MJM, RTS, JM, RCP, MCV and JGC designed the study; MJM, RTS, JM, IMC, FEF, DFL, EAR, KGF and JV conducted data collection; MJM, JGC and MCV conducted data analysis. All authors had access and participated in the interpretation of results. MJM wrote the first draft of the manuscript, reviewed initially by JGC and then by all authors who read, revised and approved the final version submitted for publication

References

1. World Health Organization. *WHO Recommendations on Antenatal Care for a Positive Pregnancy Experience*. Geneva, Switzerland; 2016.
2. Zanardo V, Mazza A, Parotto M, Scambia G, Straface G. Gestational weight gain and fetal growth in underweight women. *Ital J Pediatr*. 2016;42(1). doi:10.1186/s13052-016-0284-1
3. Rasmussen KM, Yaktine AL, Institute of Medicine (US), National Research Council (US),

For peer review only - http://bmjopen.bmj.com/site/about/guidelines.xhtml

Committee to Reexamine IOM. *Weight Gain During Pregnancy: Reexamining the Guidelines*. Washington (DC); 2009.

4. Atalah E, Castillo C, Castro R, Aldea A. Proposal of a new standard for the nutritional assessment of pregnant women. *Rev Med Chil*. 1997;125:1429-1436.

5. Brasil. Ministério da Saúde. *Manual Orientador Para Aquisição de Equipamentos Antropométricos*. 1st ed. (Secretária de Atenção a Saúde. Departamento de Atenção Básica. Coordenação Geral de Alimentação e Nutrição, ed.). Brasilia: Ministério da Saúde; 2012.

6. López LB, Calvo EB, Poy MS, del Valle Balmaceda Y, Cámera K. Changes in skinfolds and mid-upper arm circumference during pregnancy in Argentine women. *Matern Child Nutr*. 2011;7(3):253-262. doi:10.1111/j.1740-8709.2009.00237.x

7. Moller AB, Petzold M, Chou D, Say L. Early antenatal care visit: a systematic analysis of regional and global levels and trends of coverage from 1990 to 2013. *Lancet Glob Heal*. 2017;5(10):e977-e983. doi:10.1016/S2214-109X(17)30325-X

8. Das A, Saimala G, Reddy N, et al. Mid-upper arm circumference as a substitute of the body mass index for assessment of nutritional status among adult and adolescent females: learning from an impoverished Indian state. *Public Health*. 2020;179:68-75. doi:10.1016/j.puhe.2019.09.010

9. Mello LEA de M. Organização Geral do Sistema Nervoso. In: *Fisiologia Básica*. 2nd ed. Rio de Janeiro: Guanabara Koogan; 2017:131-166.

10. Morais SS, Ide M, Morgan AM, Surita FG. A novel body mass index reference range - an observational study. *Clin Sci*. 2017;11(9):698-707. doi:10.6061/clinics/2017(11)09

11. Natamba BK, Sanchez SE, Gelaye B, Williams MA. Concordance between self-reported pre-pregnancy body mass index (BMI) and BMI measured at the first prenatal study contact. *BMC Pregnancy Childbirth*. 2016;16(1):1-8. doi:10.1186/s12884-016-0983-z

12. Tang AM, Chung M, Dong K, et al. *Determining a Global Mid-Upper Arm Circumference Cutoff to Assess Malnutrition in Pregnant Women*. Washington, DC; 2016.

13. Petraro P, Madzorera I, Duggan CP, et al. Mid-arm muscle area and anthropometry predict low birth weight and poor pregnancy outcomes in Tanzanian women with HIV. *BMC Pregnancy Childbirth*. 2018;18(1). doi:10.1186/s12884-018-2136-z

For peer review only

For peer review only - http://bmjopen.bmj.com/site/about/guidelines.xhtml

416 14. Papathakis PC, Singh LN, Manary MJ. How maternal malnutrition affects linear growth and development in the offspring. *Mol Cell Endocrinol*. 2016;435:40-47. doi:10.1016/j.mce.2016.01.024

419 15. Kumar P, Sareen N, Agrawal S, Kathuria N, Yadav S, Sethi V. Screening maternal acute malnutrition using adult mid-upper arm circumference in resource-poor settings. *Indian J Community Med*. 2018;43(2):132-134. doi:10.4103/ijcm.IJCM_248_17

422 16. Sultana T, Karim MN, Ahmed T, Hossain MI. Assessment of Under Nutrition of Bangladeshi Adults Using Anthropometry: Can Body Mass Index Be Replaced by Mid-Upper-Arm-Circumference? *PLoS One*. 2015;10(4):e0121456. doi:10.1371/journal.pone.0121456

426 17. Endeshaw M, Abebe F, Worku S, Menber L, Assress M, Assefa M. Obesity in young age is a risk factor for preeclampsia : a facility based case-control study, northwest Ethiopia. *BMC Pregnancy Childbirth*. 2016;16(237). doi:10.1186/s12884-016-1029-2

429 18. Black RE, Victora CG, Walker SP, et al. Maternal and child undernutrition and overweight in low-income and middle-income countries. *Lancet*. 2013;382(9890):427-451. doi:10.1016/S0140-6736(13)60937-X

432 19. Maria A, Menezes B, Oliveira PD, et al. Associations between growth from birth to 18 years, intelligence, and schooling in a Brazilian cohort. *Am J Clin Nutr*. 2020;nqaa047. doi:10.1093/ajcn/nqaa047

435 20. Park S, Sappenfield WM, Bish C, Salihu H, Goodman D, Bensyl DM. Assessment of the Institute of Medicine recommendations for weight gain during pregnancy: Florida, 2004-2007. *Matern Child Heal J*. 2011;15(3):289-301. doi:10.1007/s10995-010-0596-5

438 21. Leary S, Fall C, Osmond C, et al. Geographical variation in relationships between parental body size and offspring phenotype at birth. *Acta Obstet Gynecol Scand*. 2006;85(9):1066-1079. doi:10.1080/00016340600697306

441 22. Instituto Brasileiro de Geografia e Estatistica- IBGE. Mapa de Densidade Demográfica de 2010. Densidade Demografica. https://censo2010.ibge.gov.br/noticias-censo.html?view=noticia&id=1&idnoticia=2501&busca=1&t=ibge-lanca-mapa-densidade-demografica-2010. Published 2010. Accessed September 1, 2020.

445 23. Instituto Brasileiro de Geografia e Estatistica- IBGE. Mapa densidade demográfica.

For peer review only - http://bmjopen.bmj.com/site/about/guidelines.xhtml

Instituto Brasileiro de Geografia - IBGE. https://brasilemsintese.ibge.gov.br/territorio/densidade-demografica.html. Published 2010. Accessed September 1, 2020.

24. Cecatti JG, Souza RT, Sulek K, et al. Use of metabolomics for the identification and validation of clinical biomarkers for preterm birth: Preterm SAMBA. *BMC Pregnancy Childbirth*. 2016;16(1):212. doi:10.1186/s12884-016-1006-9

25. Souza RT, Cecatti JG, Costa ML, et al. Planning, implementing, and running a multicentre preterm birth study with biobank resources in Brazil: The preterm samba study. *Biomed Res Int*. 2019;2019. doi:10.1155/2019/5476350

26. Brasil. Miniistério da Saude. *Orientações Para a Coleta e Análise de Dados Antropométricos Em Serviços de Saúde : Norma Técnica Do Sistema de Vigilância Alimentar e Nutricional - SISVAN / Ministério Da Saúde, Secretaria de Atenção à Saúde, Departamento de Atenção Básica.* 1st ed. (Secretaria de Atenção à Saúde. Departamento de atenção Básica., ed.). Brasilia; 2011.

27. Pinho-Pompeu M, Paulino DSM, Morais SS, Crubelatti MY, Pinto Silva JLE, Surita FG. How to classify BMI among pregnant adolescents? A prospective cohort. *Public Health Nutr*. 2019;22(2):265-272. doi:10.1017/S1368980018002768

28. Fleiss JL. *Statistical Methods for Rates and Proportions*. 2nd ed. New York: John Wiley & Sons, Ltd; 1981.

29. Fletcher RW. *Clinical Epidemiology: The Essentials*. 4th ed. Philadelphia: Lippincott Williams & Wilkins; 2005.

30. Cohen J. Weighted kappa: Nominal scale agreement provision for scaled disagreement or partial credit. *Psychol Bull*. 1968;70(4):213-220. doi:10.1037/h0026256

31. von Elm E, Altman DG, Egger M, Pocock SJ, Gøtzsche PC, Vandenbroucke JP. The strengthening the reporting of observational studies in epidemiology (STROBE) statement: Guidelines for reporting observational studies. *Int J Surg*. 2014;12(12):1495-1499. doi:10.1016/j.ijsu.2014.07.013

32. Fakier A, Petro G, Fawcus S. Mid-upper arm circumference: A surrogate for body mass index in pregnant women. *South African Med J*. 2017;107(7):606-610. doi:10.7196/SAMJ.2017.vi7.12255

For peer review only - http://bmjopen.bmj.com/site/about/guidelines.xhtml

33. Bari A, Sultana N, Mehreen S, Sadaqat N, Imran I, Javed R. Patterns of maternal nutritional status based on mid upper arm circumference. *Pakistan J Med Sci*. 2020;36(3):382-386. doi:10.12669/pjms.36.3.1331

34. T.Khadivzadeh. Mid upper arm and calf circumferences as indicators of nutritional status in women of reproductive age. *La Rev Sante Mediterr Orient*. 2002;8(2002):612-618.

35. Benítez Brito N, Suárez Llanos JP, Fuentes Ferrer M, et al. Relationship Between Mid-Upper Arm Circumference and Body Mass Index in Inpatients. *PLoS One*. 2016;11(8):1-10. doi:10.1371/journal.pone.0160480

36. Assefa N, Berhane Y, Worku A. Wealth status, mid upper arm circumference (MUAC) and antenatal care (ANC) are determinants for low birth weight in Kersa, Ethiopia. *PLoS One*. 2012;7(6):e39957. doi:10.1371/journal.pone.0039957

37. Christian P, Lee SE, Angel MD, et al. Risk of childhood undernutrition related to small-for-gestational age and preterm birth in low- and middle-income countries. *Int J Epidemiol*. 2013;42(5):1340-1355. doi:10.1093/ije/dyt109

38. Ornoy A. Prenatal origin of obesity and their complications: Gestational diabetes, maternal overweight and the paradoxical effects of fetal growth restriction and macrosomia. *Reprod Toxicol*. 2011;32(2):205-212. doi:10.1016/j.reprotox.2011.05.002

39. Ververs M, Antierens A, Sackl A, Staderini N, Captier V. Which Anthropometric Indicators Identify a Pregnant Woman as Acutely Malnourished and Predict Adverse Birth Outcomes in the Humanitarian Context? *PLoS Curr*. 2013. doi:10.1371/currents.dis.54a8b618c1bc031ea140e3f2934599c8

40. Brasil. Ministério da Saude. *Orientações Para a Coleta e Análise de Dados Antropométricos Em Serviços de Saúde : Norma Técnica Do Sistema de Vigilância Alimentar e Nutricional - SISVAN*. (Ministério da Saúde. Departamento de Atenção Básica. Secretaria de Atenção a Saúde, ed.). Brasília: Ministério da Saúde; 2011.

41. Mohanty C, Prasad R, Srikanth Reddy A, Ghosh JK, Singh TB, Das BK. Maternal anthropometry as predictors of low birth weight. *J Trop Pediatr*. 2006;52(1):24-29. doi:10.1093/tropej/fmi059

42. Van Tonder E, Mace L, Steenkamp L, Tydeman-Edwards R, Gerber K, Friskin D. Mid-upper arm circumference (MUAC) as a feasible tool in detecting adult malnutrition. *South*

For peer review only

For peer review only - http://bmjopen.bmj.com/site/about/guidelines.xhtml

African J Clin Nutr. 2019;32(4):93-98. doi:10.1080/16070658.2018.1484622

43. National Department of Health. *Guidelines for Maternity Care in South Africa*. Republic of South Africa; 2015. doi:10.4028/www.scientific.net/AMR.1010-1012.610

44. Mahomed K, Williams MA, Woelk GB, et al. Risk factors for pre-eclampsia among Zimbabwean women: maternal arm circumference and other anthropometric measures of obesity. *Paediatr Perinat Epidemiol*. 1998;12(3):253-262. doi:10.1046/j.1365-3016.1998.00116.x

45. Ngandu CB, Momberg D, Magan A, Chola L, Norris SA, Said-Mohamed R. The association between household socio-economic status, maternal socio-demographic characteristics and adverse birth and infant growth outcomes in sub-Saharan Africa: A systematic review. *J Dev Orig Health Dis*. 2019:1-18. doi:10.1017/S2040174419000680

For peer review only

For peer review only - http://bmjopen.bmj.com/site/about/guidelines.xhtml

Table 1. Distribution of the anthropometric measures for each prenatal visit and differences along the gestation.

Characteristics	19-21 (w)	27-29 (w)	1st to 2nd visit p-value*	37-39 (w)	3rd to 1st visit p-value*
**BMI (Kg/m²)**					
Mean±SD	26.33±5.36	27.96±5.36		29.93±5.34	
25th Percentile	22.50	24,20	**<0.0001**	26,30	**<0.0001**
50th Percentile	25.40	27.00		29.00	
75th Percentile	29.20	30.70		32.60	
**Total (n)**	**1,165**	**1,078**		**772**	
**Categories n (%)**					
Underweight	206 (18)	136 (13)		98 (13)	
Adequate	461 (40)	363 (34)	**<0.0001**	275 (36)	**0.0002**
Overweight	299 (26)	392 (36)		219 (28)	
Obese	199 (17)	187 (17)		180 (23)	
**MUAC (cm)**					
Mean±SD	28.71±4.64	29.32±4.54		29.66±4.35	
25th Percentile	25.20	26.00	**<0.0001**	26.50	**<0.0001**
50th Percentile	28.00	28.90		29.00	
75th Percentile	31,50	32,00		32,00	
**Total (n)**	**1,139**	**934**		**770**	
**Weight (kg)**					
Mean±SD	67.95±14.69	72.43±14.64		77.65±14.,75	
25th Percentile	57.60	62.33	**<0.0001**	67.50	**<0.0001**
50th Percentile	65.40	70.00		75.10	
75th Percentile	75.70	80.08		85.88	
**Total (n)**	**1,165**	**936**		**772**	

* ANOVA test for repeated measures. Chi square test for BMI categories. From the 3,015 MUAC measures, 8 values were considered a deviation for those seen at the second visit, the mean of the two closest measures recorded was used.

For peer review only

For peer review only - http://bmjopen.bmj.com/site/about/guidelines.xhtml

Table 2. Results of the diagnostic properties of MUAC screening for maternal nutritional status

Categories	MUAC Cutoff (cm)	Sensitivity % (95% CI)	Specificity % (95% CI)	PPV%	NPV%	Likelihood ratio (LR+)	Likelihood ratio (LR-)	ACC%	n
**First visit (19-21)**									**1138**
Underweight	<25.75	88.06 (82.57-92.05)	65.73 (60.76-70.38)	56.91	91.46	3.0	0.14	73.31	311
Adequate	≥25.75; ≤28.10	-	-	-	-	-	-	-	281
Overweight	>28.10	72.46 (64.10-79.55)	69.58 (63.58-75.00)	55.56	82.81	2.3	0.43	70.58	180
Obese	>30.15	98.92 (95.78-99.81)	60.39 (55.75-64.85)	50.00	99.29	2.5	0	71.41	366
**Second visit (27-29)**									**1076**
Underweight	<25.75	82.09 (74.32-87.97)	77.39 (73.08-81.20)	53.14	93.26	4	0.25	78.51	207
Adequate	≥25.75; ≤28.70	-	-	-	-	-	-	-	356
Overweight	>28.70	50.21 (43.69-56.73)	84.52 (80.62-87.78)	64.67	75.05	2.5	0.63	72.14	184
Obese	>30.60	99.44 (96.47-99.97)	70.30 (66.07-74.21)	54.41	99.72	3.3	0	77.95	329
**Third visit (37-39)**									**770**
Underweight	<25.75	77.32 (67.48-84.95)	82.26 (77.44-86.25)	57.69	92.06	3.5	0.38	81.08	130
Adequate	≥25.75; ≤29.45	-	-	-	-	-	-	-	277
Overweight	>29.45	42.86 (33.36-52.88)	91.16 (87.16-94.03)	63.38	81.71	4	0.67	78.45	71
Obese	>30.25	94.74 (89.93-97.41)	67.34 (62.45-71.88)	55.48	96.75	3	0.14	75.57	292

Fisher's exact test method which was statistically significant ($p<0.05$) for all categories at three set points analyzed ($p<0.0001$). PPV: Positive Predictive Value. NPV: Negative Predictive Value. ACC: Accuracy

For peer review only

For peer review only - http://bmjopen.bmj.com/site/about/guidelines.xhtml

Box 1. Tape color suggested the screening the probability of risk levels for pregnancy complication based on four MUAC cutoffs (cm) for Brazilian pregnant women at three different gestational periods

Color	19-21 (w)	27-29 (w)	37-39 (w)	Interpretation
Red	<25.75 cm			Underweight
Green	25.75 - 28.10	25.75 - 28.70	25.75 - 29.45	Adequate
Yellow	28.11 - 30.15	28.71 - 30.60	29.46 - 30.25	Overweight
Red	>30.15	>30.60	>30.25	Obesity

- Underweight = risk of poor nutrition, indicate support of diet and nutritional counseling
- Adequate = possibly no risk of poor nutrition.
- Overweight = Alert to the need for monitoring weight gain, quality of diet and excessive calories.
- Obesity = risk for adverse outcome of conditions associated with obesity including high blood pressure, gestational diabetes mellitus and large- for-gestational age fetus.

Notes:

Abbreviations: ACC – Accuracy; ANOVA - Analysis of Variance; AUC - Area Under the Curve; BMI - Body Mass Index; CI - Confidence Interval; CNPq - Brazilian National Research Council; CONEP - National Ethics Committee for Research; DRIs - Dietary Reference Intakes; IOM - Institute of Medicine; IRB - Institutional Review Board; MUAC - Mid-Upper Arm Circumference; NCDs - Noncommunicable diseases; NPV - Negative Predictive Value; PPV - Positive Predictive Value; ROC - Receiver Operating Characteristic; SAMBA - Screening And Metabolomics in Brazil and Auckland; SGA - small-for-gestational age; WHO - World Health Organization

Figure 1. Flow chart of women participating in the study

Figure 2. Correlation between BMI and MUAC for each visit (19-21, 27-29, and 37-39 weeks). Dot color indicates the BMI classification. The size of the dots represents maternal weight (kg). Classification: measurements were taken from BMI cutoff values at each week. Rho is the linear correlation coefficient. *Statistical Spearman's correlation test.

19-21(w): 95% confidence interval= 0.8567 to 0.8855, p-value = <0.0001

27-29(w): 95% confidence interval= 0.8532 to 0.8854, p-value = <0.0001

37-39(w): 95% confidence interval= 0.8070 to 0.8522, p-value = <0.0001

For peer review only

For peer review only - http://bmjopen.bmj.com/site/about/guidelines.xhtml

Figure 3. ROC curves for different MUAC cut off values based on BMI categories and the respective Area Under the Curve (AUC) at three different set points during pregnancy

Figure 4. Agreements between MUAC and BMI, according to MUAC categories by elected cutoff values.

In color, the classification is inside boxes (MUAC and BMI). Lines show the MUAC cutoff for each gestational age. The result of the degree of concordance of Cohen's Kappa according to classification at three set points are illustrated below. *Kappa agreement test

19-21(w): Kappa = 0.787, z=24.5, p-value = <0.0001

27-28(w): Kappa = 0.768, z=26.6, p-value = <0.0001

37-38(w): Kappa = 0.765, z=21.5, p-value = <0.0001

For peer review only

For peer review only - http://bmjopen.bmj.com/site/about/guidelines.xhtml

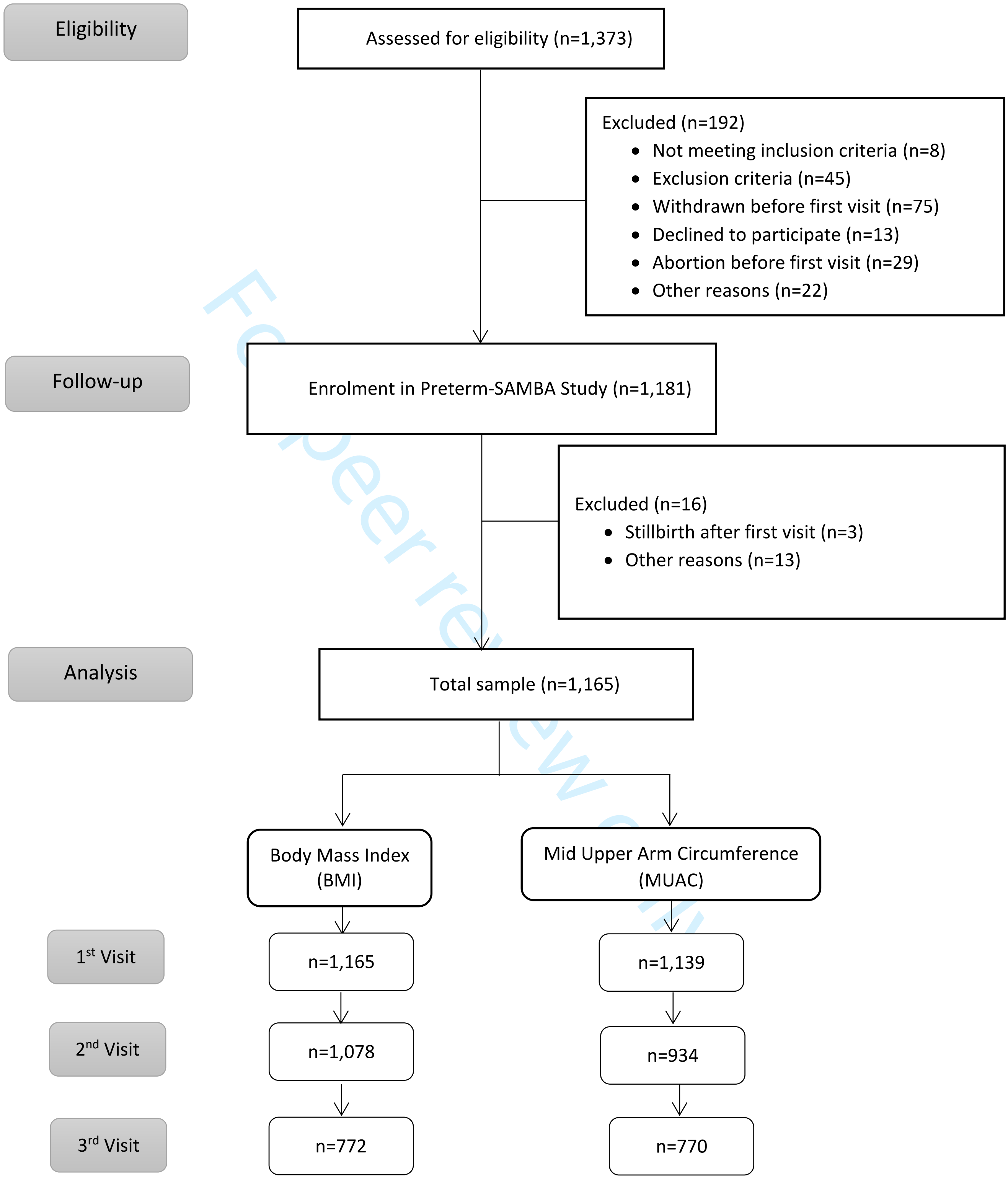
Eligibility
Assessed for eligibility (n=1,373)
Excluded (n=192)
Not meeting inclusion criteria (n=8)
Exclusion criteria (n=45)
Withdrawn before first visit (n=75)
Declined to participate (n=13)
Abortion before first visit (n=29)
Other reasons (n=22)
Follow-up
Enrolment in Preterm-SAMBA Study (n=1,181)
Excluded (n=16)
Stillbirth after first visit (n=3)
Other reasons (n=13)
Analysis
Total sample (n=1,165)
Body Mass Index (BMI)
Mid Upper Arm Circumference (MUAC)
1st Visit
n=1,165
n=1,139
2nd Visit
n=1,078
n=934
3rd Visit
n=772
n=770

19–21(w)

n=1,138

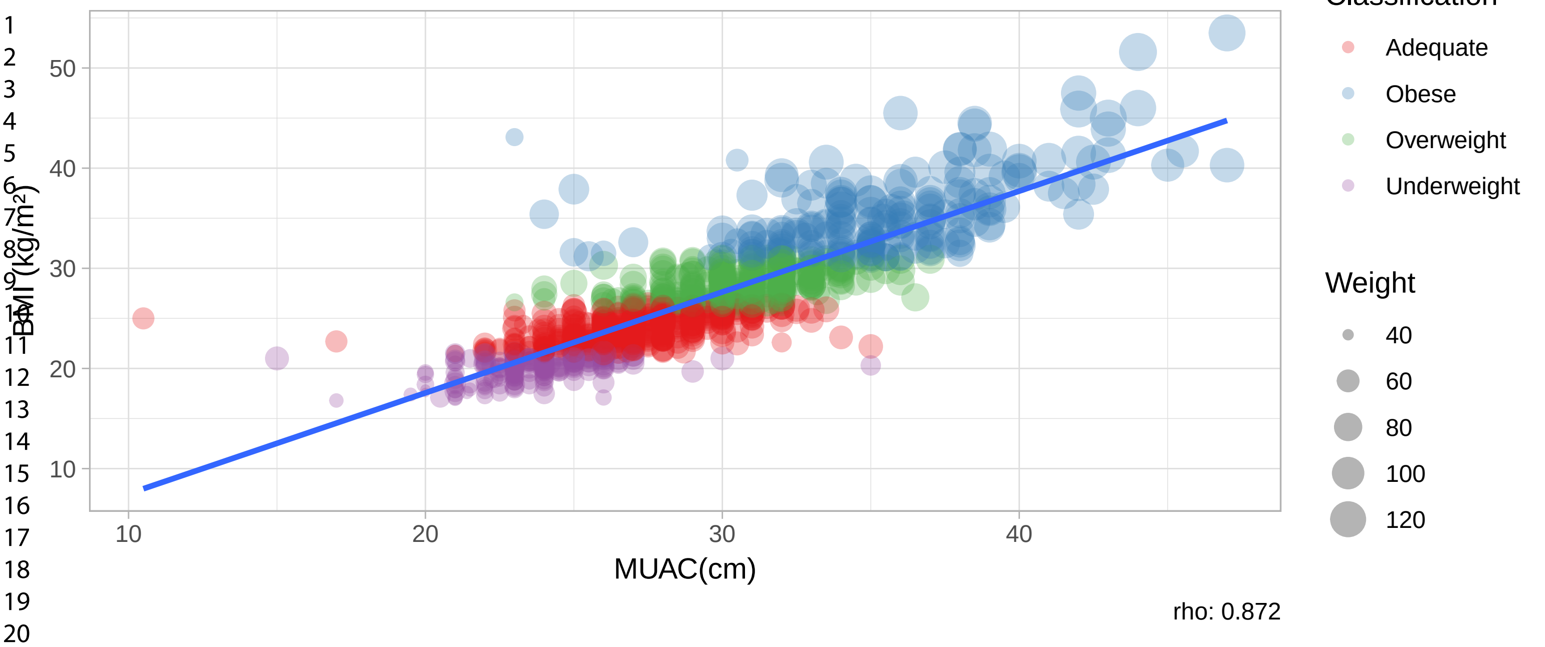

27–29(w)

n=934

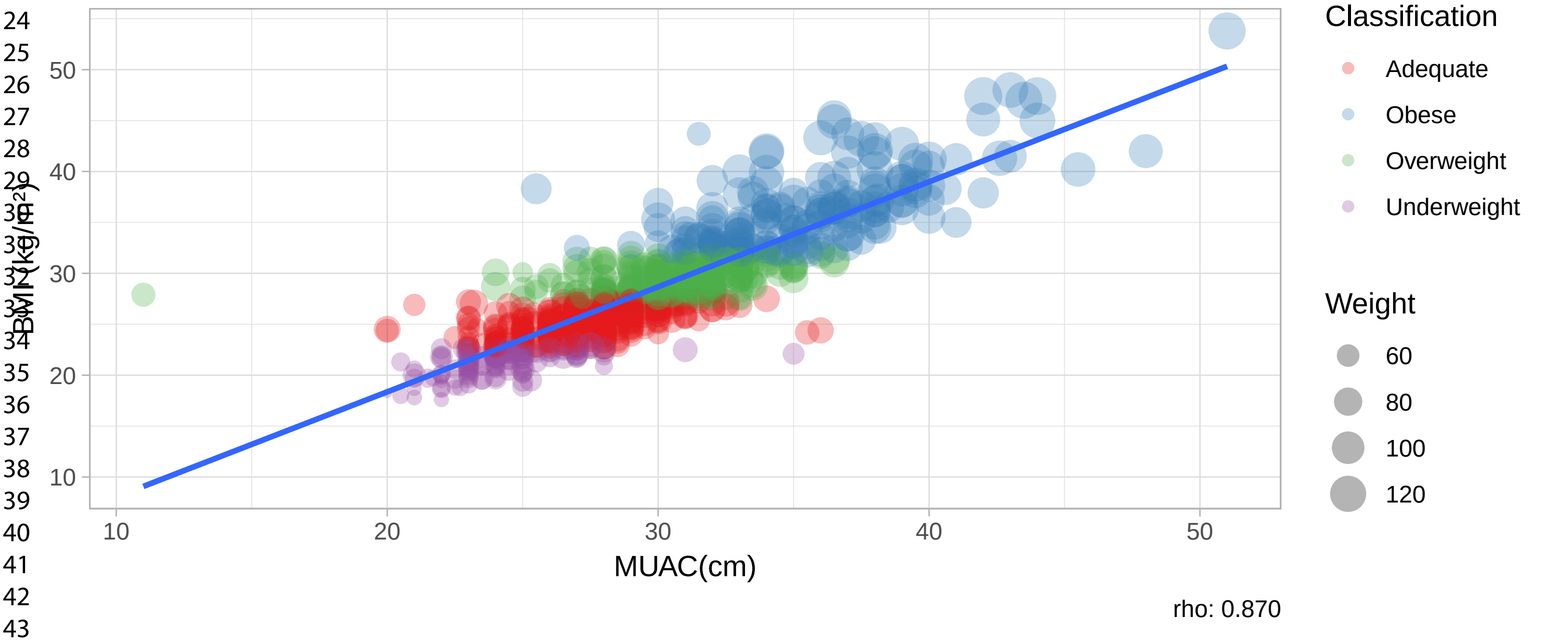

37–39(w)

n=770

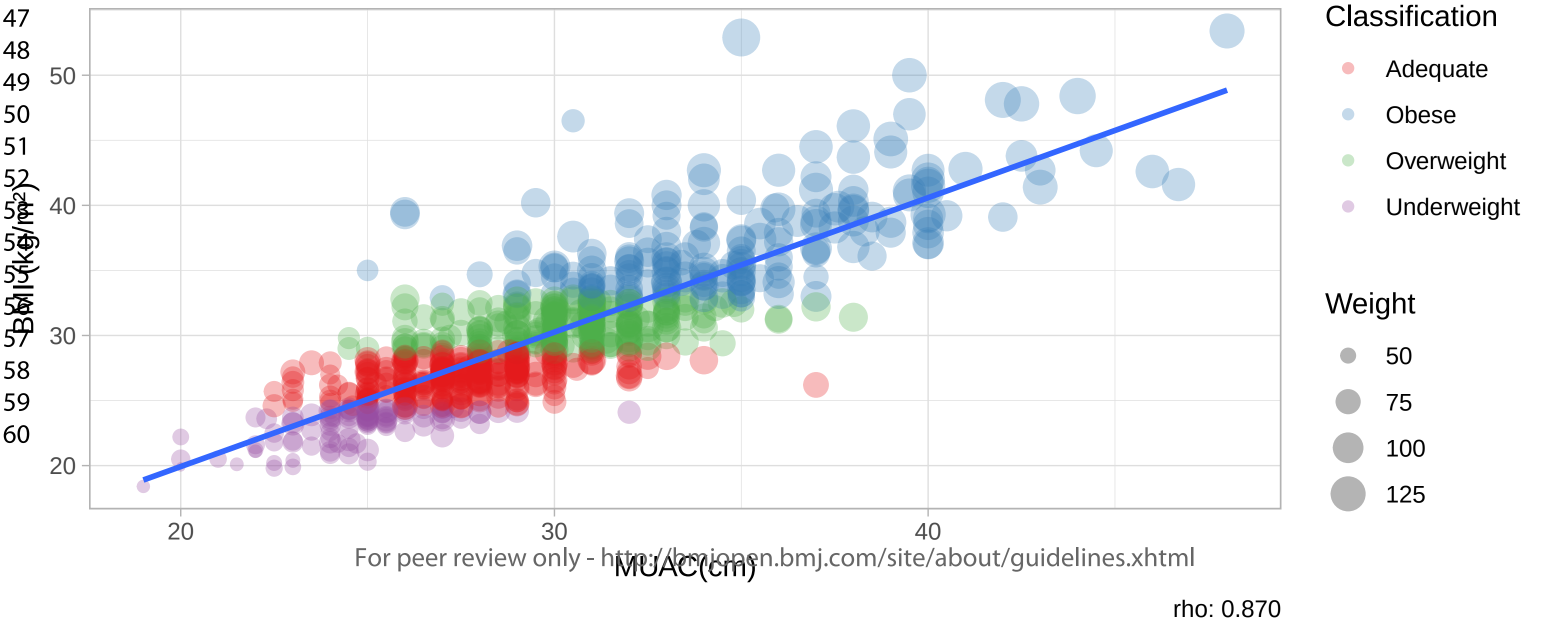

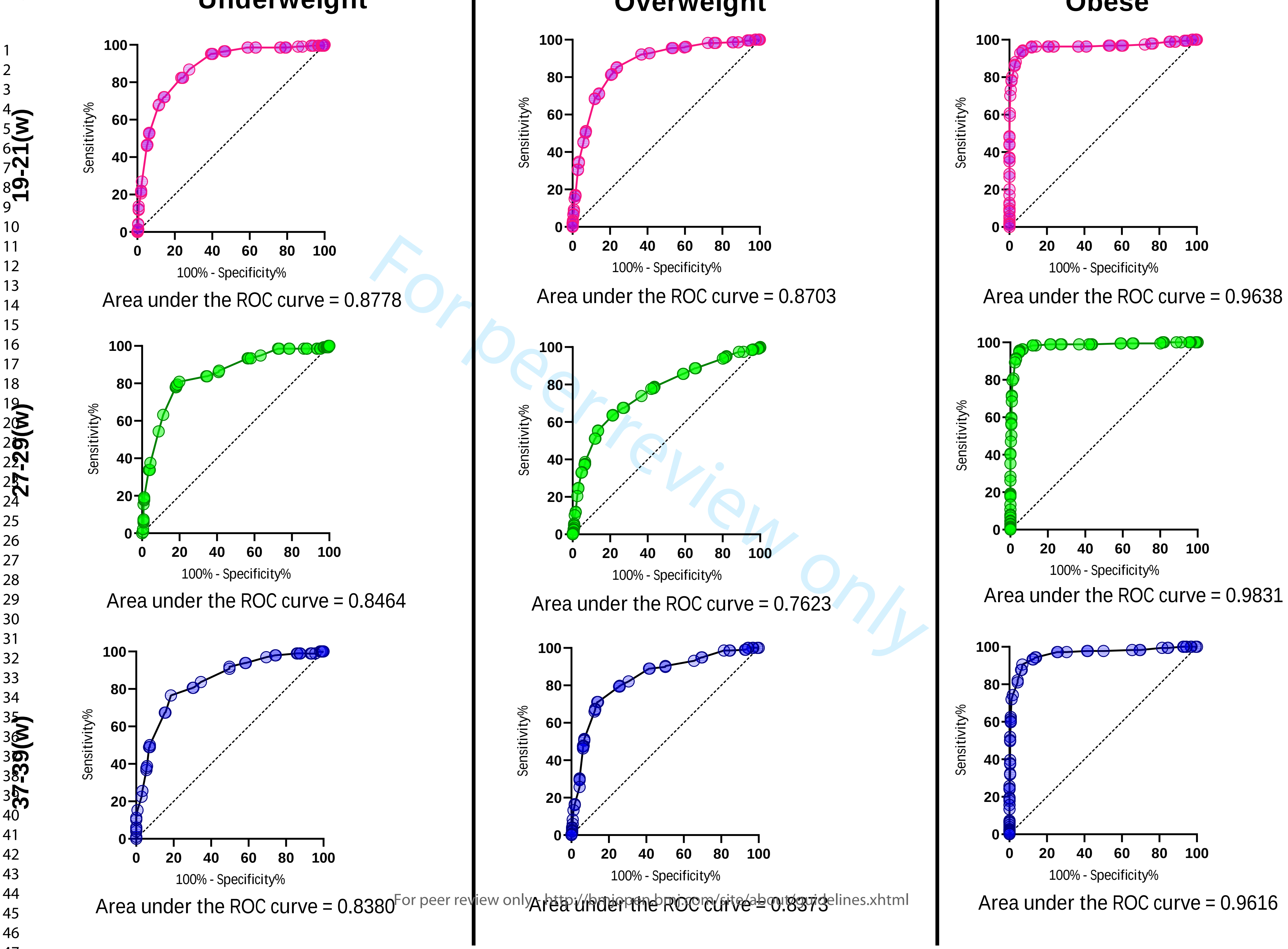

For peer review only

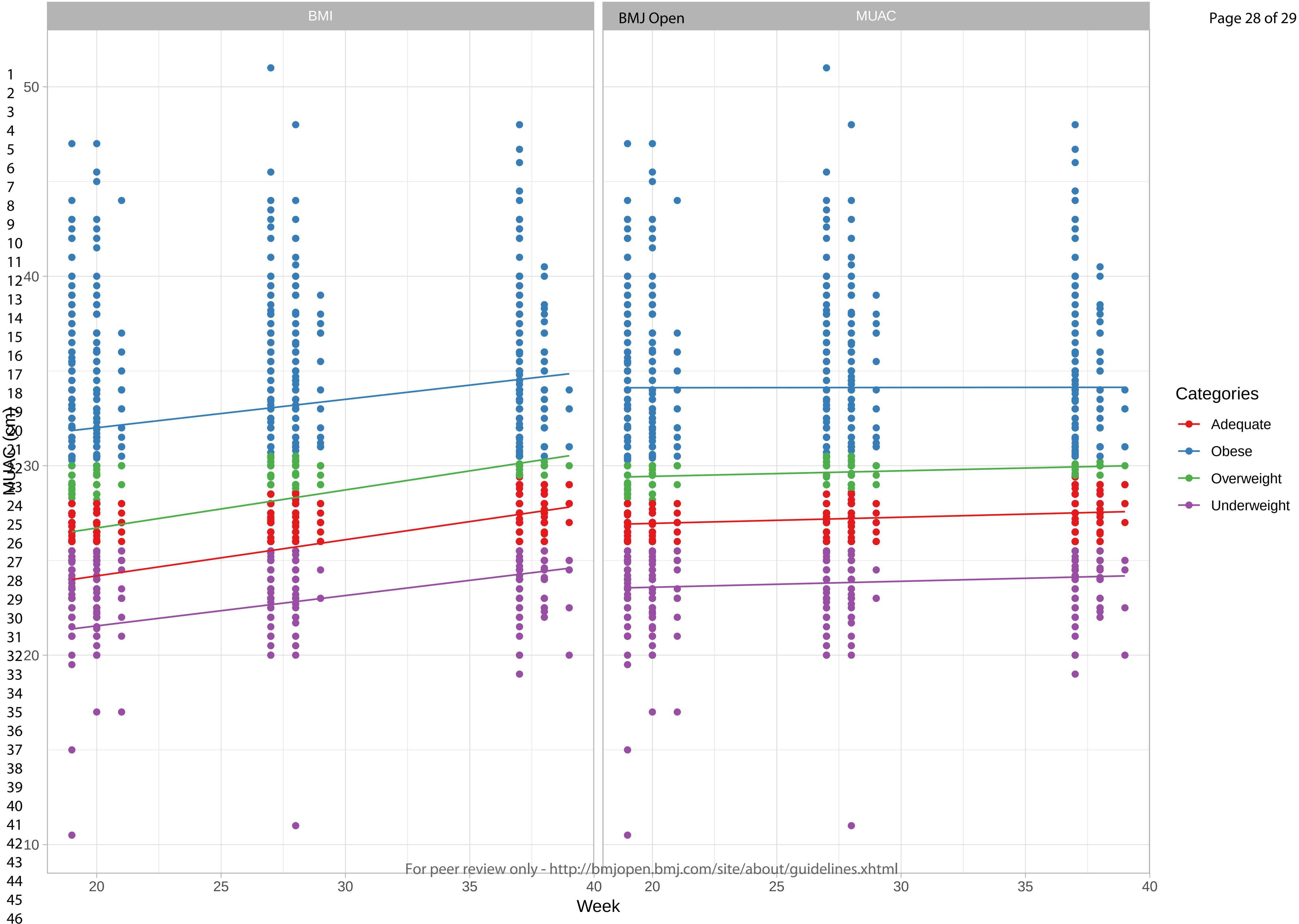
BMI
MUAC
MUAC (cm)
50
40
30
20
10
20
25
30
35
40
Week
Categories
Adequate
Obese
Overweight
Underweight

STROBE Statement—Checklist of items that should be included in reports of ***cohort studies***

	Item No	Recommendation	Page No
**Title and abstract**	1	(*a*) Indicate the study's design with a commonly used term in the title or the abstract	2
		(*b*) Provide in the abstract an informative and balanced summary of what was done and what was found	2
**Introduction**			
Background/rationale	2	Explain the scientific background and rationale for the investigation being reported	3
Objectives	3	State specific objectives, including any prespecified hypotheses	4
**Methods**			
Study design	4	Present key elements of study design early in the paper	4
Setting	5	Describe the setting, locations, and relevant dates, including periods of recruitment, exposure, follow-up, and data collection	4 Figure 1
Participants	6	(*a*) Give the eligibility criteria, and the sources and methods of selection of participants. Describe methods of follow-up	4,5
		(*b*) For matched studies, give matching criteria and number of exposed and unexposed	-
Variables	7	Clearly define all outcomes, exposures, predictors, potential confounders, and effect modifiers. Give diagnostic criteria, if applicable	4,5
Data sources/ measurement	8*	For each variable of interest, give sources of data and details of methods of assessment (measurement). Describe comparability of assessment methods if there is more than one group	5
Bias	9	Describe any efforts to address potential sources of bias	6
Study size	10	Explain how the study size was arrived at	4
Quantitative variables	11	Explain how quantitative variables were handled in the analyses. If applicable, describe which groupings were chosen and why	4-6 Table 1
Statistical methods	12	(*a*) Describe all statistical methods, including those used to control for confounding	6
		(*b*) Describe any methods used to examine subgroups and interactions	-
		(*c*) Explain how missing data were addressed	4-6
		(*d*) If applicable, explain how loss to follow-up was addressed	5-6
		(*e*) Describe any sensitivity analyses	5-6
**Results**			
Participants	13*	(a) Report numbers of individuals at each stage of study—eg numbers potentially eligible, examined for eligibility, confirmed eligible, included in the study, completing follow-up, and analysed	6-7
		(b) Give reasons for non-participation at each stage	7-8
		(c) Consider use of a flow diagram	Figure 1
Descriptive data	14*	(a) Give characteristics of study participants (eg demographic, clinical, social) and information on exposures and potential confounders	6-7 Figure 4
		(b) Indicate number of participants with missing data for each variable of interest	6-8
		(c) Summarise follow-up time (eg, average and total amount)	Table 1
Outcome data	15*	Report numbers of outcome events or summary measures over time	Table 2 Figure 3 Box 1

For peer review only

Main results	16	(*a*) Give unadjusted estimates and, if applicable, confounder-adjusted estimates and their precision (eg, 95% confidence interval). Make clear which confounders were adjusted for and why they were included	Table2<br>Figure2,3
		(*b*) Report category boundaries when continuous variables were categorized	7-8<br>Figure2,3<br>Table2<br>Box1
		(*c*) If relevant, consider translating estimates of relative risk into absolute risk for a meaningful time period	-
Other analyses	17	Report other analyses done—eg analyses of subgroups and interactions, and sensitivity analyses	Figure2,3<br>Table 2
**Discussion**			
Key results	18	Summarise key results with reference to study objectives	8
Limitations	19	Discuss limitations of the study, taking into account sources of potential bias or imprecision. Discuss both direction and magnitude of any potential bias	11,12
Interpretation	20	Give a cautious overall interpretation of results considering objectives, limitations, multiplicity of analyses, results from similar studies, and other relevant evidence	8-11
Generalisability	21	Discuss the generalisability (external validity) of the study results	11,12,13
**Other information**			
Funding	22	Give the source of funding and the role of the funders for the present study and, if applicable, for the original study on which the present article is based	12

*Give information separately for exposed and unexposed groups.

Note: An Explanation and Elaboration article discusses each checklist item and gives methodological background and published examples of transparent reporting. The STROBE checklist is best used in conjunction with this article (freely available on the Web sites of PLoS Medicine at http://www.plosmedicine.org/, Annals of Internal Medicine at http://www.annals.org/, and Epidemiology at http://www.epidem.com/). Information on the STROBE Initiative is available at http://www.strobe-statement.org.

For peer review only
